# Marine Antimicrobial Peptides: Advances in Discovery, Multifunctional Mechanisms, and Therapeutic Translation Challenges

**DOI:** 10.3390/md23120463

**Published:** 2025-12-01

**Authors:** Bin Gao, Na Yang, Da Teng, Ya Hao, Jianhua Wang, Ruoyu Mao

**Affiliations:** 1 Gene Engineering Laboratory, Feed Research Institute, Chinese Academy of Agricultural Sciences, Beijing 100081, China; 821012450528@caas.cn (B.G.); tengda@caas.cn (D.T.); haoya@caas.cn (Y.H.); wangjianhua@caas.cn (J.W.); 2 Innovative Team of Antimicrobial Peptides and Alternatives to Antibiotics, Feed Research Institute, Chinese Academy of Agricultural Sciences, Beijing 100081, China; 3 Key Laboratory of Feed Biotechnology, Department of Agriculture and Rural Affairs, Beijing 100081, China

**Keywords:** marine antimicrobial peptides, resource exploration, antimicrobial mechanism, bioactivity

## Abstract

The pervasive misuse of antibiotics has precipitated a global crisis of antimicrobial resistance (AMR), epitomized by the proliferation of methicillin-resistant Staphylococcus aureus (MRSA). Marine-derived antimicrobial peptides (AMPs) have emerged as promising alternatives, exhibiting broad therapeutic potential, including antimicrobial and anticancer activities. This review summarizes recent advances in marine AMPs, encompassing resource exploration, preparation methods, and biomedical applications, while addressing challenges such as instability and limited scalability. Future perspectives emphasize rational AMPs design to enhance efficacy and safety, alongside synergistic combination strategies, underscoring the potential of marine AMPs as viable interventions against drug-resistant pathogens.

## 1. Introduction

The extensive use of traditional antibiotics has driven the emergence of multidrug-resistant bacteria, including methicillin-resistant *Staphylococcus aureus* and carbapenem-resistant *Enterobacteriaceae*, posing a critical global health threat, with projected antimicrobial resistance–related deaths exceeding 169 million between 2025 and 2050 [1]. Consequently, there is a critical exigency for novel antimicrobial strategies. Marine AMPs, cationic and amphiphilic small peptides typically comprising 20–60 amino acids with molecular weights of 2–10 kDa, exhibit broad-spectrum antimicrobial activity, thermal stability, and alkaline properties [2,3,4]. Found across bacteria, fungi, plants, insects, amphibians, mammals, and marine species, AMPs are integral to innate immunity, providing defense against pathogenic microbes and, in some cases, inhibiting tumor cell proliferation [5,6]. Their cationic nature enables selective interaction with negatively charged bacterial membranes, while minimal interactions with zwitterionic eukaryotic membranes reduce host cytotoxicity [7]. Moreover, the dominance of zwitterionic phosphatidylcholine and sphingomyelin in eukaryotic outer leaflets creates an electrostatic barrier, thereby minimizing non-specific AMP interactions and ensuring selective toxicity [8,9]. Collectively, these characteristics highlight the considerable potential of AMPs as next-generation antimicrobial agents. Rather than concentrating on the AMPs of a single marine species or on one specific functional facet, this review provides a marine-focused synthesis that integrates state-of-the-art multi-omics and AI-driven discovery pipelines with detailed mechanistic analysis at the level of cell wall, membrane, biofilm and intracellular targets, together with a comprehensive overview of their multifunctional activities and translational hurdles. Taken together, these elements delineate a forward-looking framework that explicitly links marine AMP discovery to their clinical and industrial deployment.

As shown in Table 1, marine AMPs can be broadly classified according to their sources: (1) Marine invertebrates, which have persisted for over 450 million years and dominate marine animal diversity, possess robust innate immune systems in which hemolymph plays a key role. They constitute major AMP sources across phyla such as Arthropoda, Mollusca, Cnidaria, Protozoa, and Echinodermata [10]. (2) The evolutionary homology of marine vertebrate AMPs with mammalian host defense peptides (e.g., cathelicidins and defensins) confers superior biocompatibility and pharmacological predictability compared to invertebrate or terrestrial analogs [11]. And extreme marine selective pressures have driven the evolution of structurally robust, multifunctional scaffolds that couple potent antimicrobial activity with critical immunomodulatory functions (inflammation suppression and wound healing), thereby offering a dual-action strategy to address the pathophysiological complexity of human infections [12]. These are primarily derived from mucus, skin, scales, liver, and immune-related tissues, serving as frontline defenses at the host-environment interface [11]. Notably, mucins such as Tilapia piscidin 4 are widely expressed in mucosal barriers, contributing to innate immunity [13]. (3) Marine microbial AMPs evolved under high-salinity, low-temperature, nutrient-poor, and highly competitive conditions, yielding unique natural products for microbial competition, defense, or host protection. (4) Algal AMPs are highly diverse in structure and often chemically modified, such as through non-standard amino acids or lipid attachments, which improve their stability and activity. Algal lectins and PBPs exhibit clear antimicrobial and antifungal effects, suggesting their potential as therapeutic agent.

Occupying 71% of the planet, the oceans constitute a chemically privileged reservoir of marine AMPs, shaped by extreme salinity, hydrostatic pressure and temperature to yield exceptional structural diversity, including complex post-translational modifications and non-canonical amino acids [68,69,70]. Marine antimicrobial peptides thereby mediate broad-spectrum defense through pronounced mechanistic versatility, frequently combining multiple membrane-disruptive modes with defined intracellular targets and thus establishing activity profiles that are markedly distinct from those of most terrestrial analogs. This mechanistic spectrum extends beyond classical membrane lysis to encompass non-lytic pathways that target cell-wall components, nucleic acids, proteins or host immune signaling. Nonetheless, current insight is heavily biased towards cationic, α-helical peptides from a restricted taxonomic range, and the prevalent use of simplified assays, such as single-lipid model membranes or high peptide-to-lipid ratios, may exaggerate the perceived uniqueness of marine AMPs while obscuring their underlying mechanistic plasticity [71]. Evidence from more physiologically grounded studies indicates that individual marine AMPs can transition between membrane-lytic and intracellular modes in a pathogen- and context-dependent manner, underscoring the need for systematic, context-relevant validation of their mechanisms of action [71,72,73].

Marine AMPs often retain their activity under extreme environmental conditions and can selectively target drug-resistant pathogens through mechanisms that differ from many conventional antibiotics, thereby reducing the likelihood of resistance development [74]. Freshwater AMPs exhibit limitations akin to terrestrial analogs: their low-salinity adaptation compromises stability and activity under physiological conditions, and structural conservatism renders them susceptible to proteolysis [75]. Crucially, their mechanism is often restricted to lytic membrane disruption, lacking the multifunctionality required for complex infections, and the inherent toxicity of some highly active peptides severely restricts clinical translation [76]. Driven by the annual cataloging of over 2300 new species and identification of more than 15,000 pharmacologically active molecules, the global marine pharmaceutical market is projected to reach US$17.19 billion by 2028 [77,78,79]. Nguyen reported that rising demand for marine collagen in healthcare and nutraceuticals expanded the market from US$581.3 million in 2017 to US$897.5 million by 2023, with fish collagen peptides generating over US$405 million in 2020 and expected CAGR exceeding 5.5% through 2027 [80]. Marine AMPs often surpass conventional antibiotics, rapidly inhibiting pathogens while displaying low cytotoxicity; URP20 and epinecidin-1 are effective in infection control and wound healing [29,41]. Beyond antibacterial effects, they exhibit antiviral, antioxidant, anti-inflammatory, and metabolic benefits, acting via non-receptor-dependent membrane disruption, which limits resistance development [81].

## 2. Marine AMPs Resource Exploration

### 2.1. Exploration Technologies for Marine AMPs

Although AMPs are less prone to induce resistance than traditional antibiotics, horizontal transfer of AMP resistance genes may enable pathogens to acquire them, highlighting the need to evaluate risks and develop low-risk AMPs before clinical application [82].

In the early stages of research on AMPs, conventional biochemical assays and bioactivity-guided fractionation techniques were utilized for the isolation and identification of AMPs from crude extracts. These peptides have been shown to exhibit microbial activity against a range of pathogens, including halocidin, which was discovered in the blood cells of the solitary tunicate *Halocynthia roretzi*, and arenicin, which was isolated from the coelomic cells of the marine worm *Arenicola marina* [83,84]. The predominant isolation of marine AMPs from blood samples implicates hemolymph or circulatingocytes as their primary biosynthetic origin. In marine invertebrates, the tissues are surrounded by blood cells that have the capacity to penetrate organs. Consequently, AMPs extracted from non-blood cell tissues are likely to originate from coelomic cells [85]. Furthermore, the core functional regions of ribosomal polypeptide segments have been identified in marine biological tissues, the BjRPL27_51–72_ region located at residues 51–72 in amphioxus, making it a potential lead compound for designing novel AMPs [86]. A significant proportion of marine AMPs are characterized by the presence of cysteine and post-translational modifications of ribosomes. Modifications such as the N-terminal histidine tag in functional peptide segments have been shown to facilitate the identification of highly effective and low-toxicity peptides [87,88]. The advent of molecular biology, genomics and bioinformatics has precipitated a paradigm shift in the identification of marine AMPs. Researchers utilize high-throughput screening technologies, metagenomics and transcriptomics, to more efficiently and accurately identify and characterize novel peptide segments, decipher marine biological genetic information, and uncover potential AMPs [89]. Metagenomics, by sequencing the collective DNA of microbial communities, reveals the genetic lineages of AMPs encoded by symbiotic and pathogenic microorganisms; transcriptomics analysis elucidates the dynamic regulatory mechanisms of AMP production in response to environmental signals; and by combining computational tools and predictive algorithms, the discovery process can be optimized to identify potential AMP sequences and structural motifs from relevant datasets, accelerating their discovery [90].

### 2.2. Exploring Omics Technologies

#### 2.2.1. Genomics

Recent advances in gene sequencing, driven by projects such as Global Ocean Sampling and the Tara Oceans Expedition, have enabled metagenomic analyses to explore marine AMPs resources [77]. International teams applied bioinformatics to 240 TB of marine metagenomic data, generating the Global Ocean Microbiome catalog (GOMC) with 43,100 genomes and identifying 117 novel AMPs. Following biosynthetic and experimental validation, 10 exhibited broad-spectrum activity. These peptides show distinct sequences and structures compared to known AMPs, suggesting unique mechanisms and expanding insights into global marine antimicrobial resources [91].

Through the analysis of mixed gene sequences in environmental samples, such as seawater, sediments, or biofilms, in conjunction with metagenome-assembled genomes (MAGs) technology [92,93], the genomes of individual microorganisms can be reconstructed, and the screening of AMP-encoding genes can be undertaken. The computational mining of biosynthetic gene clusters (BGCs) has facilitated the identification of numerous novel AMP candidates [94,95]. Metagenomics enables the extraction of total DNA directly from deep-sea environmental sediments, construction of gene libraries, and screening for AMP-related gene clusters. When employed in conjunction with bioinformatics tools such as antiSMASH, this approach facilitates the prediction of the biosynthetic potential of novel AMPs. Furthermore, utilizing bioinformatics prediction and analysis tools, and drawing upon existing AMP databases, the parameters of novel AMPs can be designed, synthesized, and optimized. This is of great significance for understanding the antibacterial mechanisms of AMPs, optimizing their structures to enhance activity or reduce toxicity, and more. Furthermore, the field of bioinformatics has applications beyond the screening and discovery of AMPs. Indeed, it can also be used to systematically analyze their secondary and tertiary structure-activity characteristics, including mechanisms of action and bacterial target interactions, through structural prediction and molecular simulation techniques. This provides a theoretical foundation for the rational design and functional optimization of novel peptides. CRISPR-Cas gene editing technology can also be applied to design a series of novel AMP sequences with improved antimicrobial activity and stability. Subsequently, bioinformatics methods can be used to predict and simulate the biological characteristics of the designed AMPs to ensure their excellent antimicrobial activity. In marine environments, biofilms are microbial communities attached to any submerged substrate, such as microplastics or animal viscera [96]. Biofilm bacteria, due to their unique ecological niche and metabolic characteristics, hold great potential for discovering novel marine biofilm AMPs. Bacterial strains were isolated from marine biofilms and their genomes sequenced to establish a cultivable marine biofilm bacterial library. From 713 marine biofilm strains and their nearly complete genomes, 341 candidate AMPs were screened, among which 54 exhibited significant inhibitory activity against drug-resistant bacteria with low cytotoxicity [96]. Potential AMPs-encoding genes were identified via BLAST through whole-genome scanning technology, leading to the discovery of novel crustacean AMPs in the Indian white shrimp genome [97].

#### 2.2.2. Transcriptomics

The application of transcriptomics technologies based on high-throughput sequencing and de novo genome assembly technologies without reference genomes (Trinity) has been used to perform transcriptomics sequencing on marine biological tissues under pathogenic infection or specific physiological conditions to obtain full transcript information. Such platforms as Illumina and PacBio have been utilized for this purpose. Following infection with white spot virus, there is a significant upregulation of genes related to the phenol oxidase system, lysozyme, and defensins in blood cells [98]. In addition, the Toll-like receptor and IMD signaling pathways are activated. De novo transcriptome assembly for species lacking reference genomes is carried out with RNA-seq assemblers (e.g., Trinity, rnaSPAdes, SOAPdenovo-Trans, Trans-ABySS). Reconstructed transcripts are quantified (e.g., Salmon, Kallisto) and differential expression is assessed with DESeq2 or edgeR. Putative AMP-encoding transcripts are then nominated by ORF prediction and homology/AMP-motif screening. GO and KEGG enrichment of differentially expressed genes delineates pathways and immune responses associated with AMP biology, including membrane organization and damage-response processes [99].

Transcriptome analysis revealed that Scyampcin_44-63_ in the *Scylla paramamosain* exerts antifungal activity by inhibiting the ergosterol synthesis gene *ERG11* in *Candida albicans* and inducing the apoptosis-related gene *CASP3* [19]. The functional annotation of predicted AMP genes was conducted utilizing tools such as BLAST and InterProScan. The trends in gene expression levels between qRT-PCR and RNA-seq were found to be consistent, thereby validating the reliability of the RNA-seq data through qRT-PCR results.

#### 2.2.3. Proteomics

Proteomics-based AMPs discovery integrates multi-omics approaches, combining metagenomics for candidate sequence prediction and transcriptomics for analyzing expression regulatory networks in host–microbe interactions. Machine learning methods, including support vector machines (SVM) and deep learning models, leverage AMPs databases such as APD3, DRAMP, and DBAASP to predict novel sequences and gene clusters from metagenomic data, which are subsequently validated via proteomics. High-throughput Ribo-seq enables precise identification of small open reading frames (sORFs), as demonstrated by the 341 candidate AMPs derived from 80,430 expressed sORFs, most sharing <40% similarity with known AMPs [96]. LC-MS/MS, NMR, and molecular dynamics simulations facilitate structural characterization, elucidating α-helical conformations, membrane interactions, and post-translational modifications such as phosphorylation and glycosylation [100]. HDX-MS and simulations reveal mechanisms of membrane disruption by Pardaxin (1–22) and MSI-78 (4–20) [101], while Co-immunoprecipitation coupled with mass spectrometry identifies intracellular targets, including nucleic acids and DNA gyrase inhibitors. Transcriptomic and peptidomic integration in species such as *Pterois volitans* and *Conus betulinus* has uncovered cysteine-rich AMPs, toxin-like peptides, and 466 potential AMP-derived genes, highlighting the diversity and functional potential of marine AMPs [102,103]. Despite recent advances, proteomics-driven discovery of marine AMPs remains constrained by sparse experimental validation of metagenomic and Ribo-seq predictions, the predominance of non-physiological in vitro assays and limited genomic and animal resources, which together impede robust connections between peptide sequence, structure and in vivo function.

### 2.3. Novel Techniques and Methods

The antimicrobial potency of engineered peptides is intrinsically governed by their physicochemical attributes; however, traditional optimization via site-specific insertion, deletion, or substitution remains labor-intensive, costly, and often yields only local optima [100]. While statistical approaches such as generalized linear models, partial least squares regression, and genetic algorithms have improved sequence–activity correlations, their linear assumptions frequently fail to capture complex sequence–structure–activity relationships. Recent breakthroughs in artificial intelligence (AI) and machine learning (ML) offer a transformative paradigm for AMP discovery. Deep learning architectures, including convolutional neural networks (CNNs) and bidirectional long short-term memory networks (Bi-LSTM), excel at extracting non-linear patterns and implicit features from large datasets, facilitating de novo peptide design, feature classification, and accurate activity prediction [100]. Multi-task frameworks such as SMEP integrate classification, ranking, and regression models, enabling global scanning of 500-billion-level peptide libraries with 98.2% screening efficiency, overcoming limitations of conventional local search [104]. Furthermore, generative AI combined with synthetic biology, exemplified by GANs integrated with cell-free protein synthesis (CFPS) at the Max Planck Institute, has allowed rapid validation of 500,000 theoretical sequences within 24 h, identifying 30 broad-spectrum AMPs and substantially shortening R&D cycles [105]. Tur1A represents a pioneering success of genome-mining-led discovery. Similarly, the iAMPCN framework facilitated the identification of peptides P1-P4 from oyster mucus [30], Machine learning has also proven effective in metagenomic mining, yielding K-5, K-58, and K-61 from shrimp environments [106], and Ribo-seq coupled with a CNN-BiLSTM-Attention architecture, together with ML-guided macroalgal analyses, uncovered further marine AMPs [100]. Beyond direct validation, advanced computational strategies continue to broaden the candidate pool; PseAAC-driven models have prioritized putative AMPs from marine macroalgae [107], and workflows integrating in silico proteolysis with complex network analysis have unveiled a reservoir of non-toxic ‘encrypted’ AMPs within cephalopod salivary gland proteomes [108].

Most models are trained on legacy AMP databases that are strongly biased towards short, cationic, α-helical peptides, so predictions may preferentially rediscover the same physicochemical motif while overlooking neutral, anionic or non-helical scaffolds that are increasingly recognized among marine AMPs. Future strategies leveraging deep generative models and synthetic biology are expected to enhance AMP stability, reduce toxicity, and enable rational incorporation of specific modes of action, thereby accelerating the development of candidate therapeutic peptides.

Beyond AI applications, advances in high-throughput screening have markedly enhanced marine AMP discovery. Cell membrane chromatography (CMC) leverages bacterial membranes as stationary phases to mimic AMP–pathogen interactions, with differential peaks analyzed via high-performance liquid chromatography (HPLC) to rapidly identify active peptides. Integrating microbiome big data with multi-omics approaches enables metagenomic and metatranscriptomic analyses coupled with functional annotation, facilitating the detection of AMP genes from uncultured microorganisms. Structural predictions using AlphaFold3, combined with circular dichroism (CD) spectroscopy and electron microscopy, elucidate AMPs three-dimensional conformations and mechanisms of action, such as membrane disruption, providing a structural biology foundation for activity optimization.

## 3. Antimicrobial Mechanisms of AMPs

### 3.1. Cell Wall Target

As a vanguard of next-generation alternatives to antibiotics (ATAs), marine AMPs present a promising therapeutic strategy to combat escalating AMR. Understanding their mechanisms is essential for designing new drugs. A key aspect of AMP activity involves targeting the cell wall, the structural outer layer of bacterial and fungal cells that maintains morphology and resists stress [109]. Gram-positive bacteria, with thicker cell walls (15–50 layers) rich in peptidoglycan (PGN), contrast with Gram-negative bacteria [110]. RLvCrustinVII tightly binds *V. harveyi*, *V. parahaemolyticus*, and cell wall components (Glu, LPS, PGN), promoting bacterial aggregation and enhancing hemocyte phagocytosis in the presence of Ca^2+^ [111]. AMPs inhibit bacterial growth by disrupting cell wall integrity and interfering with PGN and wall teichoic acid (WTA) biosynthesis, thereby weakening structural stability. Lipid II, a key PGN precursor, is similarly targeted by antibiotics such as vancomycin and oritavancin, which bind it to block polymerization and compromise cell wall formation [110]. ACP1 and ACP2 from *Branchiostoma floridae* exert their inhibitory effects by specifically recognizing and binding to the Lys-type peptidoglycan of *S. aureus*, thereby restricting bacterial dissemination and promoting host immune clearance without directly lysing or killing the bacteria [112]. Cysteine-containing defensins were initially regarded as membrane-active AMPs, and the oyster defensins Cg-Defh1, Cg-Defh2, and Cg-Defm inhibit peptidoglycan biosynthesis by binding to lipid II [113]. Marine AMPs generally act after rapid electrostatic adsorption that enables immediate membrane disruption, whereas in fungi the thick chitin–β-glucan cell wall imposes a prerequisite barrier that requires specific polysaccharide recognition or uptake before the peptide can reach and kill its intracellular or membrane targets. MMGP1(marine metagenome-derived peptide) achieves cell penetration via chitin binding; once internalized, it binds DNA to inhibit transcription and subsequently induces ROS generation, mitochondrial damage, and DNA degradation, leading to apoptotic *C. albicans* cell death [114].

### 3.2. Membrane Target

Marine AMPs act primarily on microbial membranes, where electrostatically driven physicochemical interactions promote peptide accumulation at the surface, increase permeability and trigger leakage of cellular contents, culminating in rapid cell death [110]. The efficacy and topology of membrane disruption are critically governed by lipid molecular geometry: bilayers enriched in quasi-cylindrical lipids such as phosphatidylcholine (PC) and many phosphatidylglycerol (PG) species form relatively curvature-resistant planar lamellae, whereas cone-shaped lysophospholipids promote positive curvature and micellization, and inverted-cone lipids diacylglycerol, phosphatidylethanolamine (PE) and cardiolipin impose negative curvature and stabilize non-lamellar or inverted hexagonal phases that lower the energetic barrier for fusion, fission and toroidal pore formation exploited by many marine AMPs [115,116]. Within these environments, conformationally flexible α-helical marine AMPs undergo peptide-to-lipid–dependent disorder-to-helix transitions that generate amphipathic helices, and shifts in peptide-to-lipid ratio from low (surface-aligned adsorption) to high (deep insertion into the hydrophobic core) drive transleaflet remodeling, marked increases in permeability and, ultimately, complete bilayer disintegration [116,117,118].

**Figure 1:**
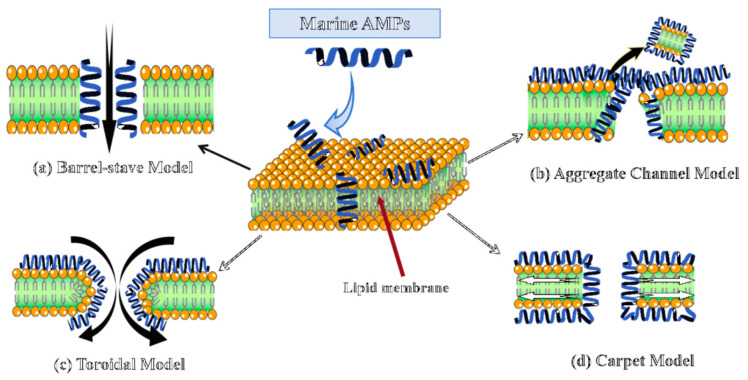
Four mechanisms of action by which marine AMPs disrupt microbial cell membranes. (**a**) Barrel model; (**b**) Aggregation channel model; (**c**) Toroidal model; (**d**) Carpet model. Cationic marine AMPs disrupt bacterial membranes via electrostatic binding to anionic lipids. The red arrow indicates the interior of the phospholipid bilayer of the cell membrane. Across the four models, AMPs markedly increase membrane permeability, provoking cell lysis and leakage of intracellular contents that culminate in bacterial death.

As shown in Figure 1, membrane disruption proceeds via four principal models: barrel-stave, aggregate channel, toroidal, and carpet [119]. While the barrel-stave model is most prominently exemplified by AMPs like Pardaxin, a lytic behavior also observed in the Piscidin and Pleurocidin families, mechanistic plasticity is prevalent, enabling a peptide like Piscidin 1 to switch its mode of action from this canonical membrane pathway against Gram-negatives to a composite membrane and intracellular targeting mechanism against Gram-positives, as dictated by the pathogen’s lipid composition [120]. In stark contrast to membrane-lytic AMPs, Myticin C executes an aggregative internal targeting mechanism that is notably pH-dependent, leveraging transcytosis to gain access to the bacterial cytoplasm for nucleic acid disruption while concurrently applying this conserved intracellular interference principle to block viral replication machinery, thereby showcasing functional adaptability across distinct pathogen classes [28]. Arenicin from *Arenicola marina* exhibits distinct mechanisms across pathogens, forming toroidal in Gram-negative membranes while inducing non-pore permeabilization in Gram-positive bacteria [112]. Likewise, Clavanin A can employ a classic carpet-like mechanism against bacterial membranes but switches to an intracellular, ROS-dependent killing pathway when targeting fungal pathogens [113]. Some peptides may also act via receptor-mediated mechanisms depending on membrane origin and lipid composition.

### 3.3. Intracellular Target

Marine AMPs traverse the plasma membrane through direct penetration, endocytic uptake, or receptor-mediated routes [121] and, upon cytosolic entry, interrupt DNA, RNA, or protein synthesis by high-affinity nucleic-acid binding. Certain marine-derived peptides, tachyplesin I and polyphemusin, transiently traverse bacterial membranes as peptide–lipid aggregates without forming pores, subsequently binding nucleic acids to inhibit DNA and RNA synthesis [72]. Other marine AMPs can penetrate cells and disrupt protein synthesis. The proline-rich Tur1A, predicted from the *Tursiops truncatus* genome, targets the ribosome, blocking translation from initiation to elongation; its direct marine homologs, retaining the -PRPX- motif, exhibit high affinity for the *E. coli* 50S subunit to halt peptide elongation [122]. Beyond nucleic acid and protein synthesis inhibition, marine AMPs may interfere with molecular chaperones to induce protein misfolding or inhibit enzymatic activity. While terrestrial proline-rich AMPs such as pyrrhocoricin, drosocin, apidaecin, and Bac7 inhibit DnaK ATPase and disrupt chaperone-mediated folding, the marine proline-rich Arasin-1 contains predicted DnaK-interacting motifs but lacks experimental validation. Structurally, ShPI-1 from *Stichodactyla helianthus*, a representative Kunitz-type protease inhibitor, specifically inhibits trypsin and chymotrypsin [123].

### 3.4. For Biofilms

Biofilms are primarily composed of proteins, polysaccharides, extracellular DNA (eDNA), and additional secreted compounds produced by adherent microbial cells, its ability to protect microorganisms from unfavorable environmental influences and enhance resistance to antibiotics [124]. EPS also stabilizes biofilm cells through a network of interactions, including cell–cell communication, gene transfer, and the formation of cooperative microaggregates [125]. *Tilapia* Hepcidin, exerts its inhibitory effect primarily on the synthesis of polysaccharides, eDNA, and proteins. Its mechanism is unique in that it targets the polysaccharide intracellular adhesion (PIA) molecule in order to reduce the quality of the extracellular matrix. This, in turn, results in a decrease in the production or degradation of the extracellular polymeric substance (EPS) of the *Staphylococcus epidermidis* biofilm matrix [110,126]. These multifaceted anti-biofilm mechanisms have significant potential for application in the treatment of chronic bacterial and fungal infections. Certain AMPs reduce biofilm formation by suppressing bacterial quorum sensing (QS), primarily through the downregulation of QS-related genes such as those in the Las and Rhl systems. The Las and Rhl networks have been shown to interact with the *Pseudomonas* quinolone signal (PQS) to form supramolecular complexes [79]. Cyclo(L-Tyr-L-Pro), isolated from *P. chrysogenum* extract, inhibited *Pseudomonas aeruginosa* biofilm formation and decreased the production of virulence factors (pyocyanin, elastase, and proteases) at 0.5mg/mL by downregulating QS genes (*lasI*, *lasR*, *rhlI*, and *rhlR*) [79].

## 4. Biological Functions of Marine AMPs

### 4.1. AMPs in Immunomodulation

Marine AMPs have emerged as multifunctional effectors that extend beyond direct pathogen elimination to the regulation of host immunity. By modulating innate immune cells, AMPs integrate antimicrobial activity with immune homeostasis. This dual function distinguishes them from conventional antibiotics, underscoring their role in maintaining the balance between immune activation and inhibition. Disruption of this balance contributes to infection, autoimmunity, and chronic inflammation, while targeted AMPs regulation offers promising therapeutic opportunities.

Marine-derived AMPs modulate inflammatory responses by balancing pro-inflammatory and anti-inflammatory signaling. Clavanin A and Clavanin-MO attenuate systemic inflammation and sepsis in murine models by downregulating pro-inflammatory cytokines such as IL-12 and TNF-α while upregulating the anti-inflammatory cytokine IL-10 [12]. In sepsis models involving both standard and multidrug-resistant bacteria, these peptides alleviated tissue pathology by repressing pro-inflammatory mediators (TNF-α, IL-1β) and augmenting anti-inflammatory responses driven by IL-10, TGF-β, and lipoxins. Oncorhynchus mykiss-derived hepcidin exhibited consistent immunomodulatory efficacy [127], and the phosvitin-derived peptide Pt5 from *Danio rerio* [128] have been shown to suppress the expression of pro-inflammatory cytokines IL-1, IL-6, TNF-α, and IFN-γ, while concurrently promoting the expression of anti-inflammatory cytokines IL-10 and IL-14, thereby enhancing overall immune function. Additionally, Thalassospiramides A and D from *Halassospira* spp. suppress LPS-induced NO production in macrophages and attenuate IL-5-driven TH2 inflammatory responses, highlighting their potential in modulating immune-mediated diseases [129]. Crus2 suppresses the ability of LPS and LTA to induce the release of IL-6, IL-1β, and TNF-α from mouse J774.1 cells [130]. EP derived from *Anguilla anguilla* potentiates macrophage-mediated immune responses by stimulating the production of NO and iNOS, increasing the secretion of TNF-α and IL-6, and activating the NF-κB and MAPK signaling pathways in a concentration-dependent manner [131]. Oral administration of the shark-derived protein hydrolysate PeptiBal™ enhances intestinal production of cytokines IL-6 and TNF-α as well as immunoglobulin IgA, subsequently increasing TGF-β and IL-10 levels and thereby indirectly mitigating gut inflammation induced by *E.coli* infection [132].

Marine AMPs possess chemotactic activity, directing immune cells to sites of infection or tissue injury and thereby facilitating effective immune responses. Fish-derived piscidins, exemplifying both antimicrobial and immunomodulatory functions, include ecPis1S, ecPis2S, ecPis3S, and ecPis4S from *Epinephelus coioides*, which not only promote chemotaxis of head kidney leukocytes—with ecPis2S exhibiting the greatest potency—but also enhance macrophage respiratory burst and phagocytosis while upregulating chemokine receptors, Toll-like receptors, T cell receptors, and pro-inflammatory cytokine expression [133]. Furthermore, it has been demonstrated for the first time that rLvCrustinIa-2, a member of the Crustin family from *Litopenaeus vannamei*, markedly enhances hemocyte chemotaxis, an activity specifically mediated by its cysteine-rich region rather than the WAP domain [134]. Calcium flux has been shown to play a critical role in cell migration and chemotaxis, and knockdown of LvCrustinIa-2 upregulates the expression of three major calcium transporters—LvNCX, LvSERCA, and LvPMCA-2—indicating that LvCrustinIa-2 may regulate calcium flux gradients during the chemotactic process. The novel antimicrobial peptide Scyreptin 1–30, derived from *Scylla paramamosain*, significantly diminished multidrug-resistant *Pseudomonas aeruginosa* load at infection sites and promoted wound repair in a murine burn model [135].

**Figure 2:**
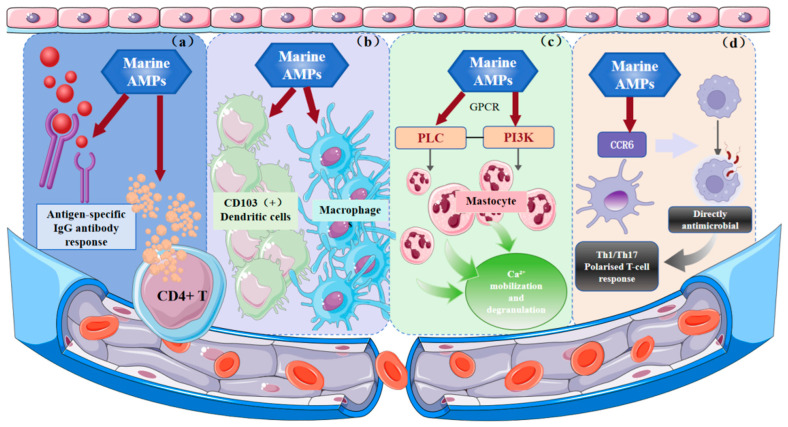
Significant immunoregulatory mechanisms of marine AMPs. (**a**) Inducing IgG antibody response; (**b**) Activating innate immune cells; (**c**) Initiation of key immune effector processes (**d**) Direct antibacterial action and polarization of T cell responses. AMPs have been shown to offer protection to the body through a series of mechanisms, including chemotactic activity, regulation of TLR ligand stimulation of host cell responses, angiogenesis, enhanced activation and differentiation of leukocytes and monocytes, and regulation of pro-inflammatory cytokine and chemokine expression.

Marine AMPs critically regulate immunity through interactions with both innate and adaptive systems. As shown in Figure 2a, in marine species, β-defensins predominate, while classical mammalian-type α-defensins are rare. By modulating immune cell distribution, β-defensins enhance host defense, and zfBD2 has been identified as a vaccine adjuvant that promotes antigen-specific IgG responses and CD4+ T cell cytokine secretion [136]. The regulatory effects of marine AMPs on innate immune cell lines are depicted in Figure 2b. Tachyplesin I enhances host defense at sublethal concentrations, stimulates macrophage activation, and can synergistically eliminate intracellular pathogens such as UPEC by combining zinc-mediated toxicity with immune enhancement [137]. As shown in Figure 2c, Pleurocidin NRC-04 activates human mast cells via GPCR-mediated PI3K, PLC, and PKC signaling, inducing chemotaxis, Ca^2+^ mobilization, and degranulation, and promoting the release of pro-inflammatory mediators to enhance innate immunity [138]. Figure 2d illustrates that pardaxin promotes differentiation of human monocyte cell lines (THP-1, U937) into phagocytosis-competent macrophages and upregulates MyD88 expression, suggesting it may modulate antigen-presenting cell function via TLR signaling, enhancing chemokine receptor-mediated interactions (e.g., CCR6 on DCs) and driving Th1/Th17-polarized T cell responses [139,140]. In summary, marine AMPs coordinate immune responses by modulating cytokines, inflammatory signaling, and immune cell recruitment and antigen presentation, underscoring their potential as therapeutic agents for immunomodulation.

### 4.2. Antiviral Properties

With advancing research, AMPs have been recognized to possess biological activities beyond antimicrobial and immunomodulatory functions. Distinct from other microorganisms, viruses are devoid of autonomous metabolic machinery and rely entirely on host cells and their metabolites to complete replication [110]. Marine AMPs exert antiviral activity through diverse mechanisms spanning the entire viral life cycle, including direct virion inactivation, inhibition of adsorption and fusion, suppression of uncoating and gene expression, and disruption of viral assembly and release. Piscidin-1 targets viral envelopes, disrupting or neutralizing enveloped particles, and has shown inhibitory effects against cholesterol-containing viruses such as SARS-CoV-2 and HIV-1 both in vitro and in vivo, highlighting its specific action on cholesterol-rich viral membranes [141]. As early as 1991, studies demonstrated that tachyplesin I inhibits HIV-1 adsorption and fusion by blocking the interaction between gp120 and coreceptors such as CXCR4, thereby suggesting a mechanism of viral entry inhibition [142]. Beyond inhibiting viral adsorption, tachyplesin has been shown to engage directly with viral particles or intracellular targets, thereby suppressing the secretion of Hepatitis B Virus (HBV) particles in Huh7 and HepG2 cells and attenuating early viral replication and antigen expression [143]. Furthermore, tachyplesin can potentiate type I interferon responses, contributing to the inhibition of aquatic viruses such as SGIV and NNV [144]. Mirabamides A–D, cyclic depsipeptides from *Siliquariaspongia mirabilis*, inhibit HIV-1 membrane fusion, preventing viral entry and subsequently disrupting assembly and release, thereby exhibiting a multi-stage antiviral mechanism targeting both early and late replication phases [145]. AVPs reported to be ~15% in some datasets, exhibiting both broad-spectrum antiviral activity and immunomodulatory functions, demonstrating significant potential as medicinal antiviral agents, particularly for enveloped viruses such as coronaviruses [110]. Current research primarily focuses on in *vitro* model development, and further studies in animal experiments and clinical trials are needed to provide more insights into their safe and effective applications and mechanisms of action.

### 4.3. Antifungal Properties

**Figure 3:**
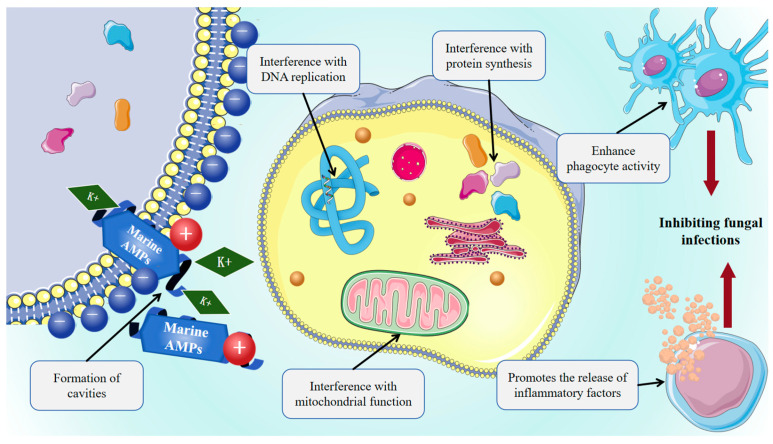
Mechanism of antifungal action of marine AMPs. Marine-derived bioactive peptides can interfere with microbial DNA or RNA replication, protein synthesis, and mitochondrial function, as well as enhance phagocyte activity and promote the release of inflammatory factors.

Marine AMPs exhibit broad-spectrum antifungal activity against clinically relevant pathogens, including yeast-like fungi such as *Candida albicans* and *Cryptococcus neoformans*, filamentous fungi such as *Aspergillus* spp. and *Mucorales,* and remain effective against azole- and echinocandin-resistant strains. This interaction promotes membrane insertion, pore formation, increased permeability, dissipation of membrane potential, K^+^ efflux, leakage of intracellular contents, and ultimately cell death. Marine AMPs can penetrate fungal membranes to disrupt the cell cycle, DNA replication, protein synthesis, and mitochondrial function, as shown in Figure 3, while also modulating host immunity by promoting inflammatory factor release or enhancing phagocyte activity, thereby exerting an indirect antifungal effect. Pleurocidin disrupts the cell membrane integrity of *Fusarium culmorum*, a producer of mycotoxins including DON, NIV, and ZEA, thereby inhibiting fungal growth [146]. Dolastatins, peptides first isolated from the *Dollabella auricularia*, exhibit strong antifungal activity against Cryptococcus neoformans and antiproliferative effects through inhibition of tubulin polymerization [147]. Epinecidin-1 enhances vaccine-induced immune responses while promoting macrophage IL-10 production, thereby facilitating the clearance of *Candida albicans* and other bacterial pathogens [41].

### 4.4. Anticancer Properties

Advanced cancer carries high mortality, while standard therapies entail severe adverse effects. Cancer cells display an elevated net negative surface charge, primarily attributed to the aberrant exposure of phosphatidylserine and elevated expression of heparan sulfate or glycosaminoglycans, making them more susceptible to targeting by cationic AMPs. Magainin induces lysis of haematopoietic and solid tumor cells via non-receptor-mediated membrane disruption with minimal lymphocyte toxicity, while Tilapia Piscidin 4 promotes bladder cancer cell apoptosis through Bcl-2/Bax modulation and ERK/SIRT1/PGC-1α activation, suppresses proliferation by downregulating cyclin D1, and has been validated in zebrafish xenografts as a potential therapeutic agent [13]. Marine AMPs exert antiviral effects primarily by targeting the viral capsid, thus blocking host cell entry or replication. Notably, cyanovirin-N, isolated from the cyanobacterium *Nostoc ellipsosporum*, suppresses HIV infection by binding to envelope glycoproteins [148]. Piscidin-1 exhibits anticancer activity against oral and oropharyngeal squamous cell carcinomas by inducing apoptosis via intrinsic and extrinsic caspase-3 pathways, elevating ER- and mitochondria-derived ROS to trigger mitochondrial dysfunction, and suppressing MMP-2/-9 in HUVECs, thereby inhibiting proliferation, migration, and angiogenesis [149]. Epinecidin-1 has been shown to inhibit LTA-induced proliferation and induce cell death in non-small cell lung cancer cells by elevating ROS levels and compromising mitochondrial function [5].

### 4.5. Antiparasitic Properties

**Figure 4:**
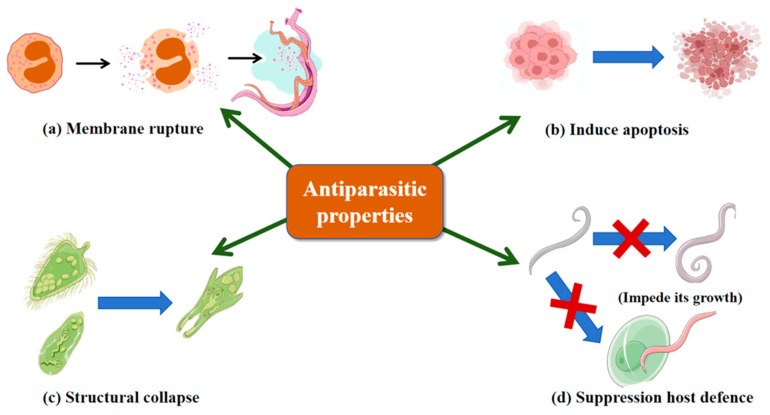
Antiparasitic properties of Marine AMPs. (**a**) Membrane rupture; (**b**) Induce apoptosis; (**c**) Structural collapse; (**d**) Suppression host defense. Blue arrows indicate promotion; blue arrows superimposed with red crosses indicate inhibition.

Parasitic worms are globally prevalent and highly transmissible, with *Plasmodium falciparum* and *Leishmania* species causing substantial morbidity and mortality. Conventional chemotherapy is limited by host toxicity and drug resistance, highlighting the need for novel antiparasitic agents. *Larimichthys crocea*-derived hepcidin-like peptide (Lc-HepL) induces membrane rupture, cytoplasmic leakage and apoptosis-like programmed cell death in *Cryptocaryon irritans* larvae (Figure 4a,b), thereby directly causing parasite mortality; it is also highly expressed in immune-relevant tissues [150]. Marine-derived AMPs exhibit potent antiparasitic activity at low concentrations. As shown in Figure 4c, tachyplesin inhibits both flagellated and non-flagellated stages of *L. donovani* via membrane disruption, limiting resistance development [151]. As illustrated in Figure 4d, jasplakinolide targets *P. falciparum* by stabilizing actin filaments through binding three adjacent actin subunits, impairing growth and host cell invasion [152], and also inhibits *Entamoeba histolytica* and *Entamoeba invadens* by inducing F-actin aggregation and dose-dependent growth suppression [153]. These findings underscore marine AMPs as promising antiparasitic agents with multi-mechanistic activity.

During early *Leishmania* infection, the complement system promotes pathogen uptake by phagocytes, enabling intracellular survival. Internalized parasites stimulate innate immune cells to release cytokines, activating adaptive responses that can be protective or contribute to tissue damage, with adaptive immune cells further participating in pathogenic mechanisms [154]. The cyclic peptide IB-01212, from the marine fungus *Clonostachys* sp. ESNA-A009, exhibits biphasic killing activity against *L. donovani* at μM concentrations, particularly inhibiting the acontous stage [59]. Malaria parasites evade host defenses by resisting lysosomal degradation and hepatocyte penetration, exploiting vesicle formation and traversing Kupffer cells and endothelial barriers to reach the erythrocytic stage. Host HO-1 facilitates parasite development during the hepatic phase through modulation of inflammatory responses [154]. These findings highlight marine-derived AMPs and host–pathogen interactions as key determinants of parasite survival and therapeutic targeting.

### 4.6. Antioxidant Properties

Reactive oxygen species (ROS) and free radicals participate in diverse metabolic processes and are implicated in the onset and progression of cancer, cardiovascular, neurological, respiratory, renal, ophthalmic, and autoimmune diseases [155]. Maintaining redox balance is essential for normal physiological metabolism, whereas excessive ROS and reactive nitrogen species (RNS) or impaired endogenous defenses result in oxidative stress. Marine-derived AMPs not only exhibit antimicrobial properties but also attenuate oxidative stress by inhibiting free radical generation. For example, Alcalase, Neutrase, and Protamex treatments of codfish skeletons yielded peptides with notable DPPH radical scavenging and iron-chelating activities [156]. Brevinin-2S Sb binds LPS and displays strong antioxidant activity, with concentration-dependent DPPH scavenging [157,158]. Similarly, antioxidant peptides from *Pangasius sutchi* and *Scomber japonicus* myofibrillar hydrolysates show high DPPH scavenging and SOD activities [159]. Hydroxyl radicals can damage DNA, disrupt membranes, and lyse erythrocytes, triggering oxidative stress and inflammation. From tuna processing waste, four novel peptides with excellent hydroxyl radical scavenging capacity have been identified, while a heptapeptide from mackerel by-products shows potent antioxidant activity and plant growth promotion [159,160]. Tuna egg–derived peptides ICRD and LCGEC display potent in vitro DPPH radical scavenging activity and safeguard HaCaT cells against UVB damage by upregulating SOD and GSH-Px [161]. Additionally, the abalone peptide ATPGEG reduces UVB-induced ROS in HaCaT cells and prevents UVB-related DNA damage [162]. Synthetic antioxidants, though effective, are limited by toxicity and carcinogenic risks. In contrast, naturally derived AMPs offer safe, sustainable alternatives, with fishery by-products providing valuable resources for functional antioxidant development. Marine peptides efficiently scavenge free radicals and prevent UV-induced damage, underscoring their potential in cosmetic applications and primary skin disease prevention.

### 4.7. Other Features

In addition to the above, AMP has anti-aging, anti-hypertensive and anti-diabetic properties. aging is characterized by a reduction in the production of extracellular matrix proteins, such as collagen, elastin, fibronectin and laminin, and an increase in their degradation. Marine fish collagen has anti-aging properties due to its skin repair and tissue regeneration capabilities. Skin aging is a multifactorial process involving both intrinsic and extrinsic mechanisms, with UV radiation being a primary cause of photoaging [163]. Pacific cod skin–derived gelatin hydrolysate mitigates UV-induced inflammation by downregulating IL-1α and TNF-α, protecting skin from radiation-induced damage and preventing photoaging [164]. Tilapia gelatin peptides protect against UV-induced skin damage and photoaging in mice, with LSGTGP effectively scavenging hydroxyl radicals to prevent UV-related injury [165]. Hyaluronic acid maintains hydration and supports skin regeneration by enhancing viscosity and reducing ECM permeability, so inhibiting its degradation is crucial for preserving skin integrity [166]. Peptides from various microalgae, including *Sukka’s algae*, *Dunaliella*, and *Nanophyllum*, have been shown to inhibit hyaluronidase activity [167]. Low-molecular-weight collagen peptides from fish scales enhance hyaluronic acid synthesis in HaCaT cells by upregulating HAS2 and downregulating HYAL1, mitigating photoaging damage [168].

Hypertension is one of the most common metabolic diseases and a significant contributing factor to conditions such as kidney failure and heart disease. The primary mechanism for its blood pressure-lowering activity is considered to be inhibiting ACE’s regulation of the renin-angiotensin system [169]. Active peptides from marine algae can inhibit ACE by blocking nucleic acid synthesis or binding, protein synthesis and membrane permeability, and by inducing cell apoptosis. The low MW (<3 kDa) components of Laminaria digitata can inhibit ACE activity by up to 75% at a concentration of 1 mg/mL [169,170]. Three ACE-inhibitory antihypertensive peptides isolated from the ovaries of *Trichiurus lepturus* demonstrated marked activity, highlighting their therapeutic potential [171].

Type 2 Diabetes Mellitus (T2DM), accounting for 90–95% of cases, is characterized by insulin resistance and insufficient secretion. Current therapies have side effects, but marine peptide hydrolysates can alleviate related syndromes by regulating glucose uptake or absorption, offering a potential alternative [172]. For instance, studies have demonstrated that hydrolysates derived from the skin gelatin by-products of *Salmo salar* have been shown to enhance insulin secretion activity by means of activating the release of glucagon-like peptide-1 (GLP-1), thereby demonstrating hypoglycaemic effects in vitro models [173]. The multifunctionality of marine peptides is attributable to their structural diversity and processing strategies, and their value in multiple fields has been validated. However, there is an urgent need for advancement in the clinical translation of these findings in order to bridge the gap between laboratory research and biomedical applications in humans.

## 5. Preparation Technology of Marine AMPs

### 5.1. Natural Extraction

The field of natural peptide extraction achieved a landmark in 1921 when Frederick Banting and colleagues successfully isolated insulin from animal pancreases [174]. This development attracted significant attention to the field, and subsequently, animal-derived peptides, adrenocorticotropic hormone and calcitonin, were translated into clinical applications. Early marine peptide isolation involved tissue processing and centrifugation, precipitation, and filtration to remove debris and concentrate peptide fractions. For example, in 1999, two isoforms of myticin, a novel cysteine-rich peptide, were isolated from the hemocytes and plasma of *Mytilus galloprovincialis* [175]. Natural marine peptides are generally safe, but industrialization is limited by complex composition, low yields, scarce active ingredients, and high costs [176,177].

### 5.2. Protease Hydrolysis Method and Optimization

Enzymatic hydrolysis represents a predominant route for marine AMP acquisition, affording high specificity under mild reaction conditions and preservation of bioactivity. This process typically involves freeze-drying and grinding marine by-products (e.g., fish, shrimp), followed by hydrolysis with magnetic immobilized neutral proteases in Tris-HCl buffer under electromagnetic assistance, and concluding with magnetic separation to obtain crude peptide extracts [159]. Proteins from *Mytilus edulis* were enzymatically hydrolysed with commercial proteases, and the resulting MAMP was purified via ion-exchange, gel filtration, and HPLC, exhibiting broad-spectrum antimicrobial activity [178]. Likewise, peptides from black-barred halfbeak fish skin were generated using trypsin and pepsin, then purified and characterized, showing both antibacterial and antifungal properties [179]. However, industrial application is constrained by high enzyme costs, limited food-grade availability, and AMPs susceptibility to proteolytic degradation, resulting in poor stability and reduced bioavailability. Organic solvent extraction is rarely used due to denaturation and low efficiency [180]. Ammonium sulfate precipitation remains common but is ineffective for low-MW AMPs (<10 kDa) and, combined with dialysis, often yields unstable and low recovery rates [181].

Optimization of protease hydrolysis requires strict regulation of parameters such as temperature, pH, enzyme type, dosage, and reaction time. Incorporating magnetic immobilized neutral proteases with alternating magnetic fields enhances motility, mitigates substrate entrapment, and improves hydrolysis efficiency, thereby increasing AMPs yield and purity [182]. Primary purification can be achieved via amino-silane-modified diatomaceous earth, which selectively adsorbs cationic AMPs and enables efficient acetone elution. Magnetic separation further accelerates enzyme recovery and reduces cost [183]. Ultrafiltration, dialysis, or gel filtration refine extracts while preserving activity, with freeze-drying producing high-activity products. Additionally, chemical modifications (cyclisation, amidation, lipidation) and delivery systems (nanoparticles, liposomes, or conjugation with cell-penetrating peptides/ligands) enhance protease resistance, stability, bioavailability, and therapeutic efficacy of marine AMPs [184,185].

### 5.3. Chemical Synthesis

Marine AMPs are primarily synthesized via liquid-phase peptide synthesis (LPPS) and solid-phase peptide synthesis (SPPS), with hybrid strategies occasionally applied for pharmaceutical peptides. LPPS, first used for insulin synthesis in 1953, is suitable for shorter peptides, allowing precise control, incorporation of rare or non-natural amino acids, and post-translational modifications [186,187]. The process involves sequential C-terminal-protected amino acid coupling, amine deprotection, and repetition until chain completion, followed by purification through crystallization or chromatography [188]. LPPS also enables the production of cyclic and structurally complex peptides, making it a pivotal method for generating marine-derived AMPs with therapeutic potential [189].

SPPS pioneered by Merrifield, is a fundamental method in peptide research, though direct synthesis suffers from low efficiency, high cost, and reagent toxicity [190]. SPPS involves immobilizing the initial amino acid on resin, followed by iterative deprotection, coupling (using Fmoc/Boc and DIC/HBTU), and washing steps, with final peptides cleaved and purified via reverse-phase HPLC [191]. This approach enables the synthesis of complex marine AMPs, incorporating non-natural amino acids and cyclic structures to enhance stability, selectivity, and efficacy [192]. SPPS also facilitates the formation of novel disulfide-bonded peptides with kinetic and stability profiles consistent with established activity standards [177]. Employing SPPS, Clavanin-MO has been produced with enhanced antimicrobial efficacy and the capacity to recruit leukocytes to infection sites, thereby modulating innate immune responses [36].

### 5.4. Cell-Free Preparation

CFPS can efficiently synthesize AMPs with more than 100 amino acids and support the integration and modification of non-natural amino acids. This significantly enhances the protease resistance and in vivo efficacy of the products CFPS reconstructs in vitro enzymatic systems using cell lysates, energy substrates, and exogenous mRNA/DNA to rapidly generate high-purity peptides [193]. CFPS platforms offer distinct advantages for producing antimicrobial peptides that are challenging to express owing to intrinsic toxicity or structural instability [194]. CFPS enables on-demand production of small peptides (<50 amino acids) from freeze-dried *E. coli* extracts, as well as larger AMPs (>100 amino acids) with non-natural residues, enhancing protease resistance and in vivo efficacy [195,196]. The technology offers sterility, portability, and elimination of cold-chain requirements, providing a versatile platform for AMPs production. A study employed high-throughput CFPS to screen and synthesize multiple antimicrobial peptides from marine metagenomes, achieving efficient identification and functional evaluation of marine-derived AMPs [106].

### 5.5. Heterologous Expression

The engineering-based production of marine AMPs leverages heterologous hosts to overcome limitations of low yield, high cost, and scarce natural extracts. Target genes are identified via transcriptomic sequencing or genome mining of marine organisms and subsequently codon-optimized for host expression. Fusion tags (His-tag, GST) and rational modifications enhance solubility, reduce toxicity, and facilitate secretion and purification [189,197]. Genes are cloned into host-specific vectors, followed by transformation, induction, and either intracellular or secreted expression. Purification employs multi-step chromatogr aphy, with fusion tags cleaved if necessary, and final refinement often achieved via reverse-phase HPLC [198,199,200].

Common bacterial hosts include *E. coli*, *B. subtilis,* and *Lactococcus lactis*, with *E. coli* preferred for its well-characterized molecular mechanisms and process scalability. Recombinant pleurocidin variants, r-pleurocidin and r-pleurocidin-G, expressed in *E. coli*, demonstrated efficacy against *Salmonella typhimurium* 14028s and *E. coli* UB1005 [201]. Large-scale optimization of *E. coli* fermentation can increase yields of AMPs substantially, for instance, raising single-batch output from 100 mg to 1000 mg while reducing unit costs by 83.4% [202]. Hal18 was efficiently expressed in *E. coli* through fusion with the baculoviral polyhedrin protein, enabling high-yield recombinant production [203]. The decisive biological activity of many AMPs is dependent on C-terminal amidation, which is typically unattainable via conventional heterologous systems. The successful preparation of native C-amidated Maculatin 1.1 utilized the His_6_-SUMO-Mac1-Mxe GyrA fusion construct, establishing a key engineering paradigm for the scalable production of fully functional marine AMPs [204]. Upon heterologous expression in the *Bacillus subtilis* system, CiMAM exhibited markedly enhanced antimicrobial efficacy, displaying potent inhibitory activity against a broad spectrum of halophilic pathogens [205]. Filamentous fungi and yeasts serve as efficient hosts due to high protein output, post-translational modification capability, and low-cost cultivation [206]. GRAS-grade fungi, such as *Aspergillus niger*, *Penicillium*, and *Trichoderma reesei*, combine efficient secretion pathways with low glycosylation levels, facilitating extracellular protein purification [207]. Single-cell eukaryotes like *S. cerevisiae* and *P. pastoris* are widely used for recombinant marine AMPs production; TP4 from *Oreochromis mossambicus* and His-tagged rTurgencin A from *Arcticus ascidium* showed broad-spectrum antibacterial activity, including against drug-resistant strains [199,208]. Mammalian hosts, such as Chinese hamster ovary (CHO) cells, can perform precise post-translational modifications but are limited by high production costs; CHO cells have been employed to express recombinant chelonianin from *Penaeus monodon* for anti-inflammatory applications [209]. While insect cells proliferate faster than mammalian hosts, specialized media and potential viral contamination necessitate careful regulatory evaluation [210]. In *Chlorella*, PC-hepc from *Pseudosciaena crocea* and scygonadin from *Scylla serrata* were combined to form the Scy-hepc gene, which was effectively expressed and conferred protection against *Aeromonas hydrophila* infection, highlighting the promising application of transgenic *Chlorella* in aquaculture [211].

### 5.6. Plant Molecular Farming

Despite challenges with membrane integrity and downstream processing (DSP), plants remain a promising production platform. Integration of synthetic biology, DSP econometric modeling, and machine learning-guided molecular optimization has recently enhanced expression efficiency and AMPs yield in plant-based systems Plant Molecular Farming (PMF) utilizes transgenic plants as scalable bioreactors for vaccines, antibodies, and therapeutic peptides, providing cost-effective production with minimal pathogen contamination [212]. Chloroplast transformation further stabilizes expression by mitigating transgene silencing and preventing pollen-mediated gene flow [213]. Closed systems, including plant cell suspensions and hairy root cultures compliant with GMP, enable controlled AMPs synthesis [214]. Recent advances integrating synthetic biology, DSP modeling, and machine learning-guided molecular optimization have significantly enhanced plant-based expression efficiency and yields of AMPs [215,216]. A *Chlorella vulgaris* chloroplast system enabled *rbcL* promoter-driven expression of codon-optimized Piscidin-4, linked via a polycistronic cassette to the endogenous ribosome binding site [217].

## 6. Marine AMPs Application Areas

### 6.1. AMPs in the Food Industry

Public concern over food safety remains significant, as risks are present across the entire supply chain—from cultivation and breeding to production, processing, packaging, storage, and distribution. Excessive use of conventional antibiotics in feed and animal husbandry has led to critical problems, including bacterial resistance and drug residues. Resistant bacteria pose a dual threat: immediately by endangering human health, and over time by disseminating resistance genes through the food chain, thereby indirectly compromising public health [218]. Clavanins have been reported to exhibit broad-spectrum antimicrobial activity against 13 foodborne pathogens, with minimum inhibitory concentrations ranging from 3 to 50 μg/mL, underscoring their potential application as natural agents for food preservation [219].

Marine-derived AMPs show strong antibacterial activity and are promising for food preservation. They inhibit spoilage microorganisms and oxidative degradation, thereby extending shelf life. Li [220] demonstrated that epinecidin-1 suppressed *Botrytis cinerea* in peaches, while Dong [27] reported that mytimacin-4 combined with chitosan, prolonged pork shelf life by 3–4 days at 4 °C through bacterial inhibition and reduced total Volatile Base Nitrogen (TVB-N) and thiobarbituric acids (TBARS). Bi [221] incorporated Sm-A1 from *Scophthalmus maximus* into a PVA/chitosan hydrogel, which effectively preserved salmon for 14 days by slowing bacterial growth, volatile nitrogen accumulation, and texture loss. Given the high water, protein, and PUFA content of seafood, AMPs offer effective strategies against spoilage. Their mechanisms include quorum sensing interference and ROS induction, with minimal risk of resistance. In milk preservation, ACWWP1 from *Coilia mystus* showed strong anti-*S. aureus* activity, reducing counts by 4.8 log CFU/mL within 24 h and sustaining inhibition over 72 h [47]. Similarly, LCWAP, derived from *Larimichthys crocea* lactic acid protein, exhibited an MIC of 15.6 µg/mL. Milk treated with 2 × MIC LCWAP displayed significant bacterial reduction within 24 h, and by day seven *S. aureus* was undetectable, compared with 9.2 log CFU/mL in controls, underscoring its potential for milk preservation [44].

### 6.2. AMPs for Agricultural Applications

In agriculture, aquaculture, and livestock farming, antibiotic feed additives have contributed to resistance, increasing the severity of once-treatable diseases. By contrast, marine AMPs used as feed additives inhibit harmful microorganisms, extend feed shelf life, suppress pathogens in aquatic animals, and enhance immunity. Studies have shown that CgPep33, derived from the enzymatic hydrolysate of Crassostrea gigas, exhibits strong antifungal activity, effectively controlling Botrytis cinerea-induced grey mold in postharvest strawberries [222].

Marine AMPs strengthen non-specific immune defenses; for instance, those from *Penaeus vannamei* are synthesized in haemolymphocytes and migrate to infection sites via chemotaxis, forming immune barriers that inhibit bacterial growth [223]. SKL17-2 from yellow croaker selectively suppresses *Pseudomonas plecoglossicida*, reducing visceral white nodule disease by 68.5% without disrupting intestinal lactobacilli [224]. Sp PR-AMP1 from mud crabs inhibits *Vibrio campbellii*, preventing acute hepatopancreatic necrosis disease [225]. Excessive use of antibiotic additives disrupts the balance of the animal gut microbiota and leaves residues in the body, thereby compromising the quality and safety of animal products. Marine AMPs also show thermal stability during feed pelleting. Examples such as astacidin, crustins, hydramacin, and pom-1 improve fish growth by enhancing serum SOD activity, intestinal protease activity, and villus structure, thereby reinforcing antioxidant defense, immunity, and nutrient absorption [226]. Synergistic interactions with functional additives further optimize feed efficiency. Moreover, the administration of 10 mg/kg of rScy-hepc as a feed additive for *Larimichthys crocea* has been demonstrated to specifically activate key growth regulatory pathways in the liver, including the GH-Jak2-STAT5-IGF1 signaling axis, the PI3K-Akt pathway, and the extracellular signal-regulated kinase/mitogen-activated protein kinase (Erk/MAPK) cascade. This, in turn, has been shown to significantly improve the specific growth rate and muscle protein deposition efficiency of individuals [227].

### 6.3. AMPs for Environmental Applications

AMPs can be used as marine antifouling materials through structural modification and functional optimization. Direct modification, such as introducing D-amino acids, metal-binding peptides or photoreactive groups, can greatly improve their stability and anti-biofilm activity. For instance, AMPs derived from *Chionoecetes opilio* containing D-amino acids can prevent the initial formation of biofilms, whereas zwitterionic peptides exhibit broad-spectrum antifouling properties by balancing charges and regulating hydrophobicity [228]. In addition, most fish farming operations typically use antibiotics to prevent disease and reduce costs. However, this practice poses a risk of antibiotic-resistant pathogens spreading through wastewater, which can lead to severe environmental pollution. While laboratory results are promising, future research is needed to validate their long-term stability in complex marine environments and to optimize designs using techniques such as molecular dynamics simulations. Halocidin and its derivatives from *Halocynthia aurantium* exhibit activity against *Candida albicans* in wastewater and soil, suggesting their potential as antifungal additives in wastewater treatment [229,230].

## 7. Challenges in the Application of Marine AMPs

### 7.1. Stability and Activity Retention

The majority of marine AMPs are native L-stereoisomers, rendering them inherently susceptible to rapid proteolytic degradation and resulting in short in vivo half-lives. and environmental instability, limiting in vivo efficacy and complicating clinical translation. It also exhibit poor metabolic stability and limited intestinal epithelial permeability [231]. Marine-derived AMPs, such as tachyplesins and pardaxins, are rapidly degraded by serum and intracellular proteases, resulting in short in vivo half-lives and limited therapeutic efficacy. Tachyplesin I is markedly degraded by extracellular proteases from *Pseudomonas aeruginosa* UV-3, with a 60% loss of antimicrobial activity within 2 h and detectable peptide fragments by LC-MS, demonstrating that marine AMPs are rapidly degraded in vivo, resulting in short half-lives and limited efficacy [232]. GE33, derived from *Pardachirus marmoratus*, self-assembles into stable peptide hydrogels at neutral pH but dissociates into monomers or oligomers under acidic conditions, while retaining therapeutic potential against *Helicobacter pylorin* [233].

Marine AMPs frequently bind non-specifically to negatively charged biomolecules or cell surfaces, including serum proteins, DNA, mucins, and glycolipids, prior to pathogen interaction [234]. Their activity and stability are influenced by sequence length, hydrophobicity, charge, amphiphilicity, secondary structure, and specific residues. Although these interdependent factors lack a clear quantitative relationship with bioactivity, they remain crucial for the rational design and synthesis of artificial AMPs [235]. Marine AMPs activity is highly sensitive to environmental factors, particularly temperature, with high heat causing structural disruption and marked activity loss. For example, pleurocidin SF1 from Pseudopleuronectes americanus is optimal at 15–20 °C, but elevated temperatures unwind its α-helix, reducing membrane-disruptive efficacy [45]. Highly hydrophobic AMPs, including pleurocidin SF1, may aggregate in aqueous solutions, diminishing effective concentrations despite amphiphilic α-helical membrane-binding properties.

Among these obstacles, protease susceptibility and off-target binding likely represent the most immediate barriers to systemic use. Encouragingly, lessons from a small number of optimized marine AMPs suggest that cyclisation, D-amino acid substitution and lipidation can improve half-life without abolishing activity, pointing to clear, testable routes for next-generation scaffold design.

### 7.2. Toxicity and Hemolytic

The cationic nature of marine AMPs drives antibacterial activity but may also cause non-specific cytotoxicity, including hemolysis, with limited toxicological data available [236]. For instance, CC34 shows low hemolytic activity in mouse and chicken erythrocytes (19.22% and 12.65%, respectively); however, the hemolytic potential of most marine AMPs remains poorly characterized, and interspecies variability may affect safety evaluations [237]. Moreover, the in vivo distribution, half-life and excretion pathways of AMPs are not well understood. Although novel marine cyclic peptides such as cereusitin B exhibit anti-biofilm activity, their potential toxicity to organs following long-term exposure is unclear [238]. The complexity of their mechanisms of action makes predicting toxicity challenging. While interacting with bacterial cell membranes, some marine AMPs may also directly bind to host cells or interact with host components, such as the extracellular matrix and cell membranes, thereby disrupting normal host cell function. Excessive suppression of immune regulatory activity may weaken the host’s immune response [239]. Plicatamide, from hemocytes of *Styela plicata*, exhibits antibacterial activity against MRSA and hemolytic effects on human erythrocytes [240]. Tachyplesin exhibits cytotoxicity toward normal lung fibroblasts (WI-38) that is only marginally lower than that observed in cancer cells. Nicomicins, isolated from *Nicomache minor*, demonstrated cytotoxic effects on both HeLa cancer cells and normal human fibroblasts, and elicited 10% hemolysis in human erythrocytes [241].

### 7.3. Production Costs and Scale-Up

The prohibitive production costs of marine AMPs constitute a formidable barrier to their commercial translation, particularly for high-dose or long-term applications. While synthetic aminoglycosides can be produced for ~$0.80/g, solid-phase synthesis of AMPs ranges from $50–400/g [242]. Natural marine AMPs occur at low concentrations, and conventional extraction and purification methods—such as gel filtration, ion exchange, and reverse-phase chromatography—are laborious, costly, and often yield low recovery, particularly for low-molecular-weight peptides [243,244]. Organic solvent extraction further risks peptide inactivation [245]. These challenges have prompted the development of cost-effective strategies, including short synthetic peptides, analogs, optimized purification protocols, and de novo design targeting key functional motifs, aiming to enhance yield, retain activity, and improve the economic feasibility of marine AMP-based therapeutics.

### 7.4. Pharmacokinetic Activity

Marine AMPs have garnered attention for their broad-spectrum antibacterial activity, but their clinical translation is limited by inherent defects and complex drug-tissue interactions. When administered systemically, AMPs have a short half-life due to rapid proteolytic degradation in the bloodstream, increased clearance by the liver and kidneys, and sensitivity to physiological salt concentrations. Furthermore, their poor intestinal mucosal permeability restricts oral administration [246,247]. However, by leveraging rational design and computer modeling, it is possible to enhance selectivity and reduce the likelihood of unintended effects. To improve the activity of drugs, methylation modifications can be used to stabilize secondary structures, increase the content of cationic residues, and strengthen interactions with microbial membranes. Designing heteropeptides or dimers to fuse functional domains is also an effective strategy.

According to the DRAMP database, despite their broad therapeutic potential, discrepancies between vitro and in vivo activities have hindered the clinical translation of several marine AMPs, including Neuprex, due to insufficient efficacy [248]. Short peptides are rapidly eliminated via plasma protein binding and renal filtration following oral or intravenous administration, exhibiting limited permeability across biological barriers—including the blood–brain barrier and fibrotic or infection-associated tissues—thus requiring advanced delivery strategies (nanocarrier-based, transdermal, or localized systems) to improve bioavailability and target site penetration [249]. Certain natural peptides and their derivatives may trigger anti-drug antibodies or hypersensitivity, and the limited knowledge of their long-term immunological and toxicological effects remains a key obstacle to regulatory approval, requiring extensive toxicity and sensitization evaluation [250].

### 7.5. Resources and Regulations

Large-scale harvesting of natural products raises ecological concerns, and marine-derived compounds face regulatory and ethical constraints, including CITES and conservation laws. Patra [251] noted that limited sample availability, seasonal and ecological constraints, and the absence of regulatory guidance on quality, immunogenicity, and environmental risks have hindered the industrial development of marine AMPs as “new chemical entities.” As of September 2025, only three Parks Australia permits for collecting marine organisms for AMP research have been approved, each requiring ecological impact assessment, with an average 14.7-month review period, delaying subsequent preclinical studies [252].

## 8. Prospects for Marine AMPs

### 8.1. Drug Development Potential

The first FDA-approved marine-derived drug was ω-conotoxin MVIIA (Ziconotide) from cone snail venom, used for pain relief, followed by the first marine-derived anticancer agent, *ecteinascidin* 743 (Yondelis) from *Ecteinascidia turbinate* [253]. Other approved marine-derived drugs include eribulin mesylate, Lovaza, Vascepa, and Epanova [254]. Currently, 23 marine-derived compounds are in clinical development (Phases I–III), with 13 additional candidates in various trial stages and numerous others in preclinical evaluation. Six approved therapeutics are marine natural product derivatives, including plitidepsin, DMXBA (GTS-21), and hemiasterlin (E7974) in Phases III, II, and I, respectively, while compounds such as chrysophaentin A, phenethylamine, and floridosides are in preclinical testing [254]. These developments underscore the significant potential of marine AMPs for future drug approval.

Marine AMPs are polymers with excellent biocompatibility and biodegradability, offering potential for new drug development. It has been established that a variety of α-amino acids and N-carboxylic acid hydrides can be synthesized into different peptides via a process known as Ring-Opening Polymerisation (ROP). Furthermore, these peptides can be modified with functional groups and bioactive agents, thus ensuring that they meet specific requirements. By adjusting the composition, sequence, and degree of polymerisation of peptides, and employing different techniques, peptides can be incorporated into nanoscale, microscale, and macroscale devices [255]. A plethora of peptides and their composites have been employed in the field of pharmaceuticals, encompassing applications such as controlled drug release, targeted gene delivery, therapeutics, and even regenerative medicine.

Marine AMPs or defensins have emerged as a promising therapeutic option for the treatment of drug-resistant bacterial infections, thereby addressing the challenges posed by the ‘post-antibiotic era’. Hydrostatin-AMP3, from *Hydrophis melanocephalus*, kills multidrug-resistant *Klebsiella pneumoniae* by disrupting membranes and intercalating DNA, with a MIC of 2 μg/mL, a therapeutic index of 32, and no resistance after 30 passages [256]. SK-Ps, derived from *Stephanonectria keithii* LZD-10-1, rapidly kills MDR bacteria by targeting membrane phospholipids, particularly phosphatidylglycerol (PG), disrupting membrane function [257]. Pleurocidin and its derivatives exhibit activity against multiple MDR bacterial strains, with MICs ranging from 2 to 256 μg/mL [258].

### 8.2. Nanotechnology Application Strategy

Marine-derived bioactive peptides offer broad-spectrum activity, but their hydrophobicity limits solubility, stability, and gastrointestinal delivery, challenges that can be addressed with nanotechnology-based systems [173,259]. Nanomaterial-based systems enhance AMP delivery by enabling efficient loading, preventing aggregation, facilitating cytoplasmic uptake, bypassing efflux pumps, and improving stability and antimicrobial efficacy with minimal toxicity [260,261]. Nanomaterialized hepcidin (rGf-Hep) from silver perch demonstrates a threefold enhancement in membrane-disruptive activity against *Vibrio parahaemolyticus* without inducing hemolysis [262,263]. The safety of nano-encapsulated marine peptides is contingent on their structural stability within the gastrointestinal tract. In the presence of digestive enzymes, strong acids, and bile salts, these peptides tend to aggregate or undergo size changes, thereby weakening their ability to penetrate biological barriers. In contrast, organic matrix nanoparticles have been shown to significantly reduce risks in comparison to metal or metal oxide systems [264]. Consequently, the employment of nano-carriers for the purpose of drug delivery serves to augment the clinical translation potential of AMPs, thereby concomitantly engendering novel strategies for intelligent biomedical applications.

## 9. Outlook and Concluding Remarks

Marine AMPs represent a promising frontier in the post-antibiotic era, with structural diversity, multifaceted mechanisms, broad-spectrum bioactivity—including antimicrobial, immunomodulatory, and anticancer effects—and low propensity for resistance, positioning them as prime candidates against AMR and unmet medical needs. Advances in exploring underexploited marine biodiversity, particularly deep-sea organisms, extremophiles, and microalgae, are expected to yield peptides with novel structural motifs and enhanced bioactivities. Integrating high-throughput sequencing, metagenomics, and deep learning-based predictive models facilitates rapid discovery and rational design of candidates with optimized antimicrobial and immunomodulatory properties. Chemical and structural modifications, such as cyclization, D-amino acid substitution, PEGylation, and lipidation, improve metabolic stability, selectivity, and pharmacokinetics, while advanced delivery platforms—including nanocarriers, hydrogels, and transdermal systems—address bioavailability and tissue-targeting challenges, enabling applications in systemic infections, wound healing, and dermatological therapies. Beyond antimicrobial activity, marine AMPs hold potential in anticancer, anti-inflammatory, cosmetic, and environmental applications. The convergence of AI-driven sequence design, automated synthesis, and high-throughput functional screening streamlines development, reduces costs, and accelerates translation, highlighting a transformative era in which computational, chemical, and biotechnological innovations collectively unlock the therapeutic and industrial potential of marine AMPs.

## Figures and Tables

**Table 1:** Marine AMPs from diverse sources.

Taxonomic	Source Classification	Peptide Name	Origin	Pharmacologic Activity	Mechanism	MIC/IC_50_	References
a. Marine Invertebrates	Arthropod	Tachyplesin I, II	*Tachypleus tridentatus*	*E. coli*, *S. aureus*, *C. albicans* and *P. pastoris*	Dual membrane disruption and intracellular argeting	0.132 μM	[14,15]
		Polyphemusins I, II	*Limulus polyphemus*	*E. coli*, *S. typhimurium* and *P. aeruginosa*	Non-lytic translocation and multi-target intracellular interference	0.098 μM	[16]
		Penaeidins	*Penaeus annamei*	*A. viridans* and *B. megaterium*	Cell-wall (chitin) targeting and membrane-active	0.3–2.5 μM	[17,18]
		Scyampcin_44–63_	*Scylla paramamosain*	*S. aureus*, *S. epidermidis*, *L. monocytogenes* and *A. baumannii*	Ergosterol-pathway interference and apoptosis induction.	3–12 μM	[19]
		Sph_12–38_	*Scylla paramamosain*	*S. Aureus*	Membrane disruption and intracellular argeting	3 μM	[20]
		Crustins	*Carcinus maenas*	*P. aeruginosa*, *V. alginolyticus* and *M. luteus*	Cell-wall targeting and secondary membrane perturbation	1.5–49.6 μM	[21]
	Annelid	Capitellacin	*Capitella teleta*	*S. aureus*, *B. subtilis* and *E. cloacae*	Membrane permeabilization and cell lysis	0.125–16 µM	[22]
		Perinerin	*Perinereis aibuhitensis*	*E. coli*, *P. aeruginosa*, *A. hydrophila* and *P. vulgaris*	Membrane permeabilization and leakage	1.98–39.68 μM	[23]
		Hedistin	*Hediste diversicolor*	*V. alginolyticus* and *M. luteus*	Membrane disruption	0.8–1.6 μM	[24]
	Mollusc	Mytilin	*Mytilus edulis*	*F. oxysporum* and *S. aureus*	Cell-wall precursor binding and secondary membrane damage	0.6–1.2 μM	[25,26]
		Mytimacin-4	*Mytilus galloprovincialis*	*E. coli*, *L. monocytogenes*, *P. aeruginosa* and *S. typhimurium*	Membrane disruption	4.18 μM	[27]
		Myticin C	*Mytilus galloprovincialis*	*E. coli*	Membrane disruption and intracellular argeting	4–32 μM	[28]
		URP20	*Crassostrea hongkongensis*	*E. coli*, *V. parahaemolyticus* and *C. albicans*	Membrane permeabilization (non-specific lytic)	0.5–5 μM	[29]
		P1–P4	*Crassostrea gigas*	*E. coli*, *S. aureus*, *and V. parahaemolyticus*	Membrane permeabilization	0.012 μM	[30]
		OctoPartenopin	*Octopus vulgaris*	*S. Aureus*, *P. aeruginosa* and *C. albicans*	Membrane interference and biofilm inhibition	15.6–62.5 μM	[31]
		Octominin	*Octopus minor*	*C. albicans*	Membrane disruption	1–8 μM	[32]
		Mytichitin-CB	*Mytilus coruscus*	*B. Subtilis* and *S. aureus*	Chitin-binding cell-wall targeting with secondary membrane disruption	<5 μM	[33]
	Cnidarian	Aurelin	*Aurelia aurita*	*E. coli* and *L. monocytogenes*	Membrane disruption	3.20–9.47 μM	[34]
		Damicornin	*Pocillopora damicornis*	*B. megaterium*, *S. aureus*, *M. luteus* and *V. shiloi*	Disrupting cytoplasmic membrane and causing lysis	1.25–20 µM	[35]
	Protochordate	Clavanin A	*Styela clava*	*S. aureus*, *E. faecium* and *P. aeruginosa*	Carpet-like membrane disruption and ROS-dependent intracellular killing	0.70 ± 0.3 µM	[36,37,38]
		Styelin A, B	*Styela clava*	*P. immobilis* and *P. citreus*	Membrane-lytic, causing rapid permeabilization	<0.5 µM	[39]
	Echinoderm	Strongylocins	*Strongylocentrotus droebachiensis*	*E. coli*, *L. anguillarum*, *S. aureus* and *C. glutamicum*	Membrane disruption	1.3–5 μM	[40]
b. Marine vertebrates	Osteichthyes fish	Tilapia piscidin 4	*Oreochromis niloticus (Nile tilapia)*	*V. vulnificus*, *A. hydrophila*, *P. aeruginosa* and *S. agalactiae*	Membrane permeabilization, depolarization and lysis	0.72 µM	[13]
		Epinecidin-1	*Epinephelus coioides*	*B. subtilis*, *V. parahaemolyticus* and *P. multocida*	Membrane disruption	2.68–4.01 µM	[41]
		Hepcidins	*Larimichthys crocea*	*M. lysodeikticus*, *S. aureus*, *A. hydrophila*	Membrane rupture and intracellular argeting	1.5625–3.125 μM	[42,43]
		LCWAP	*Larimichthys crocea*	*S. aureus* and *V. parahaemolyticus*	Membrane disruption	3.35 µM	[44]
		Pleurocidin SF	*Pseudopleuronectes americanus*	*E. coli* and *C. albicans*	Membrane disruption	18.75–37.5 μM	[45]
		Sm-A1-3	*Scophthalmus maximus*	*E. coli*, *S. typhimurium* and *H. alvei*	Membrane disruption	3.1–15.4 μM	[46]
		ACWWP1	*Coilia mystus*	*E. coli*, *S. aureus*	Direct membrane permeabilization and leakage	4.85 µM	[47]
		Piscidins 4	*Epinephelus coioides*	*Streptococcus* and *Lactococcus*	Membrane disruption and chemotaxis	1.18–2.35 µM	[48]
		Moronecidin	*Siniperca chuatsi*	*A. sobria* and *Y. ruckeri*	Membrane disruption	10–40 µM	[49]
		Pardaxin	*Pardachirus pavoninus*	*E. coli*	Membrane disruption	7.27 µM	[50]
	Cartilaginous fish	Kenojeinin I	*Raja kenojei*	*B. subtilis*, *E. coli*, *S. cerevisiae*	Membrane permeabilization	13.33–7.73 µM	[51]
	Marine reptiles	Cm-CATH 1-4	*Chelonia mydas*	*E. coli*, *S. dysenteriae* and *K. oxytoca*	Membrane permeabilization	1.30–5.20 µM	[52]
		TEWP	*Caretta caretta*	*E. coli*, *S. typhimurium* and *S. aureus*	Membrane disruption	2.8–5.1 µM	[53]
		Hc-CATH	*Hydrophis cyanocinctus*	*E. coli*, *K. pneumoniae* and *S. marcescens*	Rapid membrane permeabilization	0.71–2.84 µM	[54]
		Hydrostatin-AMP2	*Hydrophis cyanocinctus*	*E. coli*, *K. pneumoniae*, and *Cutibacterium acnes*	Membrane disruption and DNA binding	5.93 µM	[55]
	Marine mammals	Lip1	*Lipotes vexillifer*	*S. aureus* and *E. faecium*	Membrane disruption	0.5–16 μM	[56]
		Tur1A,B	*Tursiops truncatus*	*E. coli*	Intracellular targeting (protein synthesis)	1.2 ± 0.4 μM	[57]
	Myxini	Myxinidin	*Myxine glutinosa*	Not mention	Not mention	Not mention	[58]
c. Marine Microorganisms	Fungus	IB-01212	*Clonostachys* sp. *ESNA-A009*	*E. coli* and *L. donovani*	Intracellular targeting	7.1 ± 0.4 μM	[59]
		Cytosporones	*Leucostoma persoonii*	methicillin-resistant *S. aureus*	Enzyme inhibition (membrane disruption)	72–78 μM	[60]
		Diketopiperazines	*Nectria inventa*	*Trypanosoma brucei*	Intracellular metabolic inhibition	0.002–40 μM	[61]
	Bacteria	Bacillistatins	*Bacillus silvestris*	*S. pneumoniae* and *S. pyogenes*	Membrane depolarization and disruption	0.48–1.90 μM	[62]
		Unnarmicins A	*Photobacterium* sp. *strain MBIC06485*	*Pseudovibrio* sp.	Lipopeptide-type membrane disruptor	4.48–11.2 μM	[63]
		Ariakemicins A	*Rapidithrix* sp.	*Brevibacterium* sp., *S. aureus* and *B. subtilis*	Cell-wall targeting and membrane disruption	0.31–53.33 μM	[64]
d. Marine algae		AQ-1756 etc.	*Tetraselmis suecica*	*E. coli* and *M. luteus*	Membrane disruption	50 μM	[65]
		Lectin	*Kappaphycus striatum*	*V. alginolyticus* and *E. cloacae*	Cell-wall targeting and membrane binding	1.81–7.26 μM	[66]
		PBPs	*Hydropuntia cornea*	*H. cornea*, *A. platensis*	Membrane permeabilization and oxidative stress	0.192 μM	[67]

## Data Availability

No new data were created or analyzed in this study. Data sharing is not applicable to this article.

## References

[1] Ahmad M., Aduru S.V., Smith R.P., Zhao Z., Lopatkin A.J. (2025). The role of bacterial metabolism in antimicrobial resistance. Nat. Rev. Microbiol..

[2] Singh P.K., Sharma S., Kumari A., Korpole S. (2014). A non-pediocin low molecular weight antimicrobial peptide produced by *Pediococcus pentosaceus* strain IE-3 shows increased activity under reducing environment. BMC Microbiol..

[3] Waghu F.H., Barai R.S., Gurung P., Idicula-Thomas S. (2016). CAMPR3: A database on sequences, structures and signatures of antimicrobial peptides. Nucleic. Acids. Res..

[4] Gallardo-Becerra L., Cervantes-Echeverria M., Cornejo-Granados F., Vazquez-Morado L.E., Ochoa-Leyva A. (2023). Perspectives in searching antimicrobial peptides (AMPs) produced by the microbiota. Microb. Ecol..

[5] Yu H.H., Wu L.Y., Hsu P.L., Lee C.W., Su B.C. (2024). Marine antimicrobial peptide epinecidin-1 inhibits proliferation induced by lipoteichoic acid and causes cell death in non-small cell lung cancer cells via mitochondria damage. Probiotics Antimicrob. Proteins.

[6] Suttmann H., Retz M., Paulsen F., Harder J., Zwergel U., Kamradt J., Wullich B., Unteregger G., Stockle M., Lehmann J. (2008). Antimicrobial peptides of the cecropin-family show potent antitumor activity against bladder cancer cells. BMC Urol..

[7] Panayi T., Diavoli S., Nicolaidou V., Papaneophytou C., Petrou C., Sarigiannis Y. (2024). Short-chained linear scorpion peptides: A pool for novel antimicrobials. Antibiotics.

[8] Moretta A., Scieuzo C., Petrone A.M., Salvia R., Manniello M.D., Franco A., Lucchetti D., Vassallo A., Vogel H., Sgambato A. (2021). Antimicrobial peptides: A new hope in biomedical and pharmaceutical fields. Front. Cell. Infect. Microbiol..

[9] Erdem B.M., Kesmen Z. (2022). Antimicrobial peptides (AMPs): A promising class of antimicrobial compounds. J. Appl. Microbiol..

[10] Wang S., Fan L., Pan H., Li Y., Qiu Y., Lu Y. (2023). Antimicrobial peptides from marine animals: Sources, structures, mechanisms and the potential for drug development. Front. Mar. Sci..

[11] Rakers S., Niklasson L., Steinhagen D., Kruse C., Schauber J., Sundell K., Paus R. (2013). Antimicrobial peptides (AMPs) from fish epidermis: Perspectives for investigative dermatology. J. Invest. Dermatol..

[12] Kang H.K., Lee H.H., Seo C.H., Park Y. (2019). Antimicrobial and immunomodulatory properties and applications of marine-derived proteins and peptides. Mar. Drugs.

[13] Chang C., Chang P., Lee Y., Pan C., Chang H., Wu W., Lin M., Chen C., Wen Z., Lee C. (2024). The antimicrobial peptide tilapia piscidin 4 induced the apoptosis of bladder cancer through ERK/SIRT1/PGC-1alpha signaling pathway. Probiotics Antimicrob. Proteins.

[14] Laederach A., Andreotti A.H., Fulton D.B. (2002). Solution and micelle-bound structures of tachyplesin I and its active aromatic linear derivatives. Biochemistry.

[15] Muta T., Fujimoto T., Nakajima H., Iwanaga S. (1990). Tachyplesins isolated from hemocytes of Southeast Asian horseshoe crabs (*Carcinoscorpius rotundicauda* and *Tachypleus gigas*): Identification of a new tachyplesin, tachyplesin III, and a processing intermediate of its precursor. J. Biochem..

[16] Zhang L., Scott M.G., Yan H., Mayer L.D., Hancock R.E. (2000). Interaction of polyphemusin I and structural analogs with bacterial membranes, lipopolysaccharide, and lipid monolayers. Biochemistry.

[17] Meng M., Ning J., Yu J., Chen D., Meng X., Xu J., Zhang J. (2014). Antitumor activity of recombinant antimicrobial peptide penaeidin-2 against kidney cancer cells. J. Huazhong Univ. Sci. Technolog. Med. Sci..

[18] Destoumieux D., Munoz M., Bulet P., Bachere E. (2000). Penaeidins, a family of antimicrobial peptides from penaeid shrimp (Crustacea, Decapoda). Cell. Mol. Life Sci..

[19] Zhou Y., Meng X., Chen F., Xiong M., Zhang W., Wang K. (2023). Newly discovered antimicrobial peptide scyampcin(44-63) from *scylla paramamosain* exhibits a multitargeted candidacidal mechanism in vitro and is effective in a murine model of vaginal candidiasis. Antimicrob. Agents. Chemother..

[20] Ma X., Hou L., Chen B., Fan D., Chen Y., Yang Y., Wang K. (2017). A truncated sph_12–38_ with potent antimicrobial activity showing resistance against bacterial challenge in *Oryzias melastigma*. Fish Shellfish Immunol..

[21] Guterstam P., Madani F., Hirose H., Takeuchi T., Futaki S., El Andaloussi S., Graslund A., Langel U. (2009). Elucidating cell-penetrating peptide mechanisms of action for membrane interaction, cellular uptake, and translocation utilizing the hydrophobic counter-anion pyrenebutyrate. Biochim. Biophys. Acta.

[22] Panteleev P.V., Tsarev A.V., Safronova V.N., Reznikova O.V., Bolosov I.A., Sychev S.V., Shenkarev Z.O., Ovchinnikova T.V. (2020). Structure elucidation and functional studies of a novel beta-hairpin antimicrobial peptide from the marine Polychaeta *Capitella teleta*. Mar. Drugs.

[23] Pan W., Liu X., Ge F., Han J., Zheng T. (2004). Perinerin, a novel antimicrobial peptide purified from the clamworm Perinereis aibuhitensis grube and its partial characterization. J. Biochem..

[24] Tasiemski A., Schikorski D., Le Marrec-Croq F., Pontoire-Van Camp C., Boidin-Wichlacz C., Sautiere P. (2007). Hedistin: A novel antimicrobial peptide containing bromotryptophan constitutively expressed in the NK cells-like of the marine annelid, Nereis diversicolor. Dev. Comp. Immunol..

[25] Lofgren S.E., Miletti L.C., Steindel M., Bachere E., Barracco M.A. (2008). Trypanocidal and leishmanicidal activities of different antimicrobial peptides (AMPs) isolated from aquatic animals. Exp. Parasitol..

[26] Roch P., Yang Y., Toubiana M., Aumelas A. (2008). NMR structure of mussel mytilin, and antiviral-antibacterial activities of derived synthetic peptides. Dev. Comp. Immunol..

[27] Dong B., Wang Y., Cui G., Wang Y., Lin Y., Su Z., Zhao G. (2024). In vitro antimicrobial activity of the novel antimicrobial peptide mytimacin—4 and its influence on the microbial community and quality of pork during refrigerated storage. Food Control.

[28] Domeneghetti S., Franzoi M., Damiano N., Norante R., Haifawy N.M.E., Mammi S., Marin O., Bellanda M., Venier P. (2015). Structural and antimicrobial features of peptides related to myticin c, a special defense molecule from the mediterranean mussel *Mytilus galloprovincialis*. J. Agric. Food. Chem..

[29] Mao F., Bao Y., Wong N.K., Huang M., Liu K., Zhang X., Yang Z., Yi W., Shu X., Xiang Z. (2021). Large-Scale plasma peptidomic profiling reveals a novel, nontoxic, *Crassostrea hongkongensis*-derived antimicrobial peptide against foodborne pathogens. Mar. Drugs.

[30] Song J., Liu K., Jin X., Huang K., Fu S., Yi W., Cai Y., Yu Z., Mao F., Zhang Y. (2024). Machine learning-driven discovery and evaluation of antimicrobial peptides from *Crassostrea gigas* mucus proteome. Mar. Drugs.

[31] Maselli V., Galdiero E., Salzano A.M., Scaloni A., Maione A., Falanga A., Naviglio D., Di Cosmo A., Galdiero S. (2020). OctoPartenopin: Identification and preliminary characterization of a novel antimicrobial peptide from the suckers of *Octopus vulgaris*. Mar. Drugs.

[32] Nikapitiya C., Dananjaya S.H.S., Chandrarathna H.P.S.U., De Zoysa M., Whang I. (2020). Octominin: A novel synthetic anticandidal peptide derived from defense protein of *Octopus minor*. Mar. Drugs.

[33] Liu H., Fan M., Liu H., Qi P., Zhi L. (2018). Production and function of different regions from mytichitin-1 of *Mytilus coruscus*. Fish Shellfish Immunol..

[34] Ovchinnikova T.V., Balandin S.V., Aleshina G.M., Tagaev A.A., Leonova Y.F., Krasnodembsky E.D., Men’Shenin A.V., Kokryakov V.N. (2006). Aurelin, a novel antimicrobial peptide from jellyfish *Aurelia aurita* with structural features of defensins and channel-blocking toxins. Biochem. Biophys. Res. Commun..

[35] Vidal-Dupiol J., Ladriere O., Destoumieux-Garzon D., Sautiere P., Meistertzheim A., Tambutte E., Tambutte S., Duval D., Foure L., Adjeroud M. (2011). Innate immune responses of a scleractinian coral to vibriosis. J. Biol. Chem..

[36] Silva O.N., de la Fuente-Nunez C., Haney E.F., Fensterseifer I.C.M., Ribeiro S.M., Porto W.F., Brown P., Faria-Junior C., Rezende T.M.B., Moreno S.E. (2016). An anti-infective synthetic peptide with dual antimicrobial and immunomodulatory activities. Sci. Rep..

[37] Miller A., Matera-Witkiewicz A., Mikolajczyk A., Wieczorek R., Rowinska-Zyrek M. (2021). Chemical “butterfly effect” explaining the coordination chemistry and antimicrobial properties of clavanin complexes. Inorg. Chem..

[38] Lee I.H., Cho Y., Lehrer R.I. (1997). Effects of pH and salinity on the antimicrobial properties of clavanins. Infect. Immun..

[39] Lee I.H., Cho Y., Lehrer R.I. (1997). Styelins, broad-spectrum antimicrobial peptides from the solitary tunicate, *Styela clava*. Comp. Biochem. Physiol. B-Biochem. Mol. Biol..

[40] Li C., Haug T., Styrvold O.B., Jorgensen T.O., Stensvag K. (2008). Strongylocins, novel antimicrobial peptides from the green sea urchin, *Strongylocentrotus* droebachiensis. Dev. Comp. Immunol..

[41] Chee P.Y., Mang M., Lau E.S., Tan L.T., He Y.W., Lee W.L., Pusparajah P., Chan K.G., Lee L.H., Goh B.H. (2019). Epinecidin-1, an antimicrobial peptide derived from grouper (*Epinephelus coioides*): Pharmacological activities and applications. Front. Microbiol..

[42] Wang K., Cai J., Cai L., Qu H., Yang M., Zhang M. (2009). Cloning and expression of a hepcidin gene from a marine fish (*Pseudosciaena crocea*) and the antimicrobial activity of its synthetic peptide. Peptides.

[43] Zhang J., Yan Q., Ji R., Zou W., Guo G. (2009). Isolation and characterization of a hepcidin peptide from the head kidney of large yellow croaker, *Pseudosciaena crocea*. Fish Shellfish Immunol..

[44] Yang S., Li J., Aweya J.J., Yuan Z., Weng W., Zhang Y., Liu G. (2020). Antimicrobial mechanism of *Larimichthys crocea* whey acidic protein-derived peptide (LCWAP) against *Staphylococcus aureus* and its application in milk. Int. J. Food Microbiol..

[45] Choi M., Jang H.S., Oh Y.D., Jeon Y., Kim J., Lim H.K. (2025). Characterization and antimicrobial activity of a novel pleurocidin in starry flounder (*Platichthys stellatus*). Fish Shellfish Immunol..

[46] Gonzalez Garcia M., Rodriguez A., Alba A., Vazquez A.A., Morales Vicente F.E., Perez-Erviti J., Spellerberg B., Stenger S., Grieshober M., Conzelmann C. (2020). New antibacterial peptides from the freshwater Mollusk *Pomacea poeyana* (Pilsbry, 1927). Biomolecules.

[47] Pu C., Tang W. (2017). Affinity and selectivity of anchovy antibacterial peptide for *Staphylococcus aureus* cell membrane lipid and its application in whole milk. Food Control.

[48] Noga E.J., Silphaduang U., Park N.G., Seo J.K., Stephenson J., Kozlowicz S. (2009). Piscidin 4, a novel member of the piscidin family of antimicrobial peptides. Comp. Biochem. Physiol. B-Biochem. Mol. Biol..

[49] Sun B.J., Xie H.X., Song Y., Nie P. (2007). Gene structure of an antimicrobial peptide from mandarin fish, *Siniperca chuatsi* (Basilewsky), suggests that moronecidins and pleurocidins belong in one family: The piscidins. J. Fish Dis..

[50] Thennarasu S., Nagaraj R. (1996). Specific antimicrobial and hemolytic activities of 18-residue peptides derived from the amino terminal region of the toxin pardaxin. Protein Eng..

[51] Cho S.H., Lee B.D., An H., Eun J.B. (2005). Kenojeinin I, antimicrobial peptide isolated from the skin of the fermented skate, *Raja kenojei*. Peptides.

[52] Qiao X., Yang H., Gao J., Zhang F., Chu P., Yang Y., Zhang M., Wang Y., Yu H. (2019). Diversity, immunoregulatory action and structure-activity relationship of green sea turtle cathelicidins. Dev. Comp. Immunol..

[53] Chattopadhyay S., Sinha N.K., Banerjee S., Roy D., Chattopadhyay D., Roy S. (2006). Small cationic protein from a marine turtle has beta-defensin-like fold and antibacterial and antiviral activity. Proteins.

[54] Wei L., Gao J., Zhang S., Wu S., Xie Z., Ling G., Kuang Y., Yang Y., Yu H., Wang Y. (2015). Identification and characterization of the first cathelicidin from sea snakes with potent antimicrobial and anti-inflammatory activity and special mechanism. J. Biol. Chem..

[55] Wang S., Fan L., Pan H., Li Y., Zhao X., Qiu Y., Lu Y. (2023). Identification and Characterization of a novel cathelicidin from *Hydrophis cyanocinctus* with antimicrobial and anti-Inflammatory activity. Molecules.

[56] Sola R., Mardirossian M., Beckert B., Sanghez De Luna L., Prickett D., Tossi A., Wilson D.N., Scocchi M. (2020). Characterization of cetacean proline-rich antimicrobial peptides displaying activity against ESKAPE pathogens. Int. J. Mol. Sci..

[57] Mardirossian M., Perebaskine N., Benincasa M., Gambato S., Hofmann S., Huter P., Muller C., Hilpert K., Innis C.A., Tossi A. (2018). The dolphin proline-rich antimicrobial peptide Tur1A inhibits protein synthesis by targeting the bacterial ribosome. Cell Chem. Biol..

[58] Cherniavskyi Y.K., Oliva R., Stellato M., Vecchio P.D., Galdiero S., Falanga A., Dames S.A., Tieleman D.P. (2024). Structural characterization of the antimicrobial peptides myxinidin and WMR in bacterial membrane mimetic micelles and bicelles. Biochim. Et Biophys. Acta (BBA)—Biomembr..

[59] Luque-Ortega J.R., Cruz L.J., Albericio F., Rivas L. (2010). The antitumoral depsipeptide IB-01212 kills *leishmania* through an apoptosis-like process involving intracellular targets. Mol. Pharm..

[60] Beau J., Mahid N., Burda W.N., Harrington L., Shaw L.N., Mutka T., Kyle D.E., Barisic B., Van Olphen A., Baker B.J. (2012). Epigenetic tailoring for the production of anti-infective cytosporones from the marine fungus *Leucostoma persoonii*. Mar. Drugs.

[61] Watts K.R., Ratnam J., Ang K., Tenney K., Compton J.E., Mckerrow J., Crews P. (2010). Assessing the trypanocidal potential of natural and semi-synthetic diketopiperazines from two deep water marine-derived fungi. Bioorg. Med. Chem..

[62] Pettit G.R., Knight J.C., Herald D.L., Pettit R.K., Hogan F., Mukku V.J.R.V., Hamblin J.S., Dodson M.J., Chapuis J. (2009). Antineoplastic agents. 570. Isolation and structure elucidation of bacillistatins 1 and 2 from a marine *Bacillus silvestris*. J. Nat. Prod..

[63] Oku N., Kawabata K., Adachi K., Katsuta A., Shizuri Y. (2008). Unnarmicins a and c, new antibacterial depsipeptides produced by marine bacterium *Photobacterium* sp. MBIC06485. J. Antibiot..

[64] Oku N., Adachi K., Matsuda S., Kasai H., Takatsuki A., Shizuri Y. (2008). Ariakemicins a and b, novel polyketide-peptide antibiotics from a marine gliding bacterium of the genus Rapidithrix. Org. Lett..

[65] Guzman F., Wong G., Roman T., Cardenas C., Alvarez C., Schmitt P., Albericio F., Rojas V. (2019). Identification of antimicrobial peptides from the Microalgae *Tetraselmis suecica* (Kylin) butcher and bactericidal activity improvement. Mar. Drugs.

[66] Shin E., Hwang H., Kim I., Nam T. (2011). A glycoprotein from *Porphyra yezoensis* produces anti-inflammatory effects in liposaccharide-stimulated macrophages via the TLR4 signaling pathway. Int. J. Mol. Med..

[67] Righini H., Francioso O., Di Foggia M., Quintana A.M., Roberti R. (2020). Preliminary Study on the activity of phycobiliproteins against *Botrytis cinerea*. Mar. Drugs.

[68] Bo J., Yang Y., Zheng R., Fang C., Jiang Y., Liu J., Chen M., Hong F., Bailey C., Segner H. (2019). Antimicrobial activity and mechanisms of multiple antimicrobial peptides isolated from rockfish *Sebastiscus marmoratus*. Fish Shellfish Immunol..

[69] Rajanbabu V., Chen J.Y. (2011). Applications of antimicrobial peptides from fish and perspectives for the future. Peptides.

[70] Chaturvedi P., Bhat R.A.H., Pande A. (2020). Antimicrobial peptides of fish: Innocuous alternatives to antibiotics. Rev. Aquac..

[71] Nguyen L.T., Haney E.F., Vogel H.J. (2011). The expanding scope of antimicrobial peptide structures and their modes of action. Trends. Biotechnol..

[72] Qi-Yu Z., Zhi-Bin Y., Yue-Ming M., Xiang-Yu H., Gang S., Xu-Rui C., Jun L., Melbourne U.O., Cai-Yun F. (2021). Antimicrobial peptides: Mechanism of action, activity and clinical potential. Mil. Med. Res..

[73] Kumari S., Booth V. (2022). Antimicrobial peptide mechanisms studied by Whole-Cell deuterium NMR. Int. J. Mol. Sci..

[74] Guryanova S.V., Ovchinnikova T.V. (2025). Multifaceted marine peptides and their therapeutic potential. Mar. Drugs.

[75] Zheng S., Tu Y., Li B., Qu G., Li A., Peng X., Li S., Shao C. (2025). Antimicrobial peptide biological activity, delivery systems and clinical translation status and challenges. J. Transl. Med..

[76] Su Z., Yu H., Lv T., Chen Q., Luo H., Zhang H. (2025). Progress in the classification, optimization, activity, and application of antimicrobial peptides. Front. Microbiol..

[77] Bouchet P., Kantor Y.I., Sysoev A., Puillandre N. (2011). A new operational classification of the conoidea (Gastropoda). J. Molluscan Stud..

[78] Boeuf G. (2011). Marine biodiversity characteristics. Comptes Rendus Biol..

[79] Jeong G., Khan F., Tabassum N., Cho K., Kim Y. (2024). Marine-derived bioactive materials as antibiofilm and antivirulence agents. Trends. Biotechnol..

[80] Nguyen T., Sperou N., Su P., Zhang W. (2022). Marine biorefinery: An environmentally sustainable solution to turn marine biomass and processing wastes into value-added products and profits. Biochem..

[81] Andersson D.I., Hughes D., Kubicek-Sutherland J.Z. (2016). Mechanisms and consequences of bacterial resistance to antimicrobial peptides. Drug Resist. Update.

[82] Jangir P.K., Ogunlana L., Maclean R.C. (2021). Evolutionary constraints on the acquisition of antimicrobial peptide resistance in bacterial pathogens. Trends Microbiol..

[83] Ramesh C., Tulasi B.R., Raju M., Thakur N., Dufosse L. (2021). Marine Natural Products from tunicates and their associated microbes. Mar. Drugs.

[84] Stanovova M., Gazizova G.R., Gorbushin A.M. (2023). Transcriptomic profiling of immune-associated molecules in the coelomocytes of lugworm *Arenicola marina* (Linnaeus, 1758). J. Exp. Zool. Part B.

[85] Sperstad S.V., Haug T., Blencke H., Styrvold O.B., Li C., Stensvag K. (2011). Antimicrobial peptides from marine invertebrates: Challenges and perspectives in marine antimicrobial peptide discovery. Biotechnol. Adv..

[86] Chen Y., Yi M., Wang Y., Yao L., Ji G., Gao Z. (2025). Identification of a novel antimicrobial peptide from amphioxus ribosomal protein L27. Fish Shellfish Immunol..

[87] Ahn M., Rajasekaran G., Gunasekaran P., Ryu E.K., Lee G., Hyun J., Cheong C., Kim N., Shin S.Y., Bang J. (2014). Enhancement of antibacterial activity of short tryptophan-rich antimicrobial peptide Pac-525 by replacing Trp with His (chx). Bull. Korean Chem. Soc..

[88] Ahn M., Murugan R.N., Jacob B., Hyun J., Cheong C., Hwang E., Park H., Seo J., Srinivasrao G., Lee K.S. (2013). Discovery of novel histidine-derived lipo-amino acids: Applied in the synthesis of ultra-short antimicrobial peptidomimetics having potent antimicrobial activity, salt resistance and protease stability. Eur. J. Med. Chem..

[89] Yang B., Yang H., Liang J., Chen J., Wang C., Wang Y., Wang J., Luo W., Deng T., Guo J. (2025). A review on the screening methods for the discovery of natural antimicrobial peptides. J. Pharm. Anal..

[90] Kavousi K., Bagheri M., Behrouzi S., Vafadar S., Atanaki F.F., Lotfabadi B.T., Ariaeenejad S., Shockravi A., Moosavi-Movahedi A.A. (2020). IAMPE: NMR-assisted computational prediction of antimicrobial peptides. J. Chem Inf. Model..

[91] Chen J., Jia Y., Sun Y., Liu K., Zhou C., Liu C., Li D., Liu G., Zhang C., Yang T. (2024). Global marine microbial diversity and its potential in bioprospecting. Nature.

[92] Shen H., Li Y., Pi Q., Tian J., Xu X., Huang Z., Huang J., Pian C., Mao S. (2025). Unveiling novel antimicrobial peptides from the ruminant gastrointestinal microbiomes: A deep learning-driven approach yields an anti-MRSA candidate. J. Adv. Res..

[93] Chen B., Zhang Z., Zhang Q., Xu N., Lu T., Wang T., Hong W., Fu Z., Penuelas J., Gillings M. (2023). Antimicrobial peptides in the global microbiome: Biosynthetic genes and resistance determinants. Environ. Sci. Technol..

[94] Wei B., Hu G., Zhou Z., Yu W., Du A., Yang C., Yu Y., Chen J., Zhang H., Wu Q. (2023). Global analysis of the biosynthetic chemical space of marine prokaryotes. Microbiome.

[95] Prichula J., Primon-Barros M., Luz R.C.Z., Castro I.M.S., Paim T.G.S., Tavares M., Ligabue-Braun R., D’Azevedo P.A., Frazzon J., Frazzon A.P.G. (2021). Genome mining for antimicrobial compounds in wild marine animals-associated *enterococci*. Mar. Drugs.

[96] Fan S., Qin P., Lu J., Wang S., Zhang J., Wang Y., Cheng A., Cao Y., Ding W., Zhang W. (2024). Bioprospecting of culturable marine biofilm bacteria for novel antimicrobial peptides. Imeta.

[97] Sruthy K.S., Nair A., Puthumana J., Antony S.P., Singh I.S.B., Philip R. (2017). Molecular cloning, recombinant expression and functional characterization of an antimicrobial peptide, Crustin from the Indian white shrimp, Fenneropenaeus indicus. Fish Shellfish Immunol..

[98] Lin J., Wan H., Xue H., He Y., Peng B., Zhang Z., Wang Y. (2024). Transcriptomics reveals different response mechanisms of *Litopenaeus vannamei* hemocytes to injection of Vibrio parahaemolyticus and WSSV. Comp. Biochem. Physiol. D-Genomics Proteomics.

[99] Fei Y., Wang Q., Lu J., Ouyang L., Hu Q., Chen L. (2023). New insights into the antimicrobial mechanism of LEAP2 mutant zebrafish under *Aeromonas hydrophila* infection using transcriptome analysis. Fish Shellfish Immunol..

[100] Gao Q., Ge L., Wang Y., Zhu Y., Liu Y., Zhang H., Huang J., Qin Z. (2025). An explainable few-shot learning model for the directed evolution of antimicrobial peptides. Int. J. Biol. Macromol..

[101] Lin B., Hung A., Li R., Barlow A., Singleton W., Matthyssen T., Sani M., Hossain M.A., Wade J.D., O’Brien-Simpson N.M. (2022). Systematic comparison of activity and mechanism of antimicrobial peptides against nosocomial pathogens. Eur. J. Med. Chem..

[102] Houyvet B., Bouchon-Navaro Y., Bouchon C., Corre E., Zatylny-Gaudin C. (2021). Marine transcriptomics analysis for the identification of new antimicrobial peptides. Mar. Drugs.

[103] Li R., Huang Y., Peng C., Gao Z., Liu J., Yin X., Gao B., Ovchinnikova T.V., Qiu L., Bian C. (2022). High-throughput prediction and characterization of antimicrobial peptides from multi-omics datasets of Chinese tubular cone snail (*Conus betulinus*). Front. Mar. Sci..

[104] Huang J., Xu Y., Xue Y., Huang Y., Li X., Chen X., Xu Y., Zhang D., Zhang P., Zhao J. (2023). Identification of potent antimicrobial peptides via a machine-learning pipeline that mines the entire space of peptide sequences. Nat. Biomed. Eng..

[105] Pandi A., Adam D., Zare A., Trinh V.T., Schaefer S.L., Burt M., Klabunde B., Bobkova E., Kushwaha M., Foroughijabbari Y. (2023). Cell-free biosynthesis combined with deep learning accelerates de novo-development of antimicrobial peptides. Nat. Commun..

[106] Wu K., Xu G., Tian Y., Li G., Yi Z., Tang X. (2025). Synthesis and evaluation of aquatic antimicrobial peptides derived from marine metagenomes using a high-throughput screening approach. Mar. Drugs.

[107] Caprani M., Slattery O., O’Keeffe J., Healy J. (2020). Identification of antimicrobial peptides from macroalgae with machine learning. Proceedings of the International Conference on Practical Applications of Computational Biology & Bioinformatics.

[108] Aguero-Chapin G., Marrero-Ponce Y., Castillo-Mendieta K., Antunes A. (2024). Unveiling encrypted antimicrobial peptides from cephalopods’ salivary glands: A proteolysis-driven virtual approach. ACS Omega.

[109] Mohanram H., Bhattacharjya S. (2014). Resurrecting inactive antimicrobial peptides from the lipopolysaccharide trap. Antimicrob. Agents. Chemother..

[110] Li X., Zuo S., Wang B., Zhang K., Wang Y. (2022). Antimicrobial mechanisms and clinical application prospects of antimicrobial peptides. Molecules.

[111] Hu J., Li S., Lv Q., Miao M., Li X., Li F. (2022). Characterization of the Dual Functions of *LvCrustinVII* from *Litopenaeus vannamei* as antimicrobial peptide and opsonin. Mar. Drugs.

[112] Li J., Li Y., Fan Z., Chen S., Yan X., Yue Z., Huang G., Liu S., Zhang H., Chen S. (2021). Two amphioxus ApeC-Containing proteins bind to microbes and inhibit the TRAF6 pathway. Front. Immunol..

[113] Schmitt P., Wilmes M., Pugniere M., Aumelas A., Bachere E., Sahl H., Schneider T., Destoumieux-Garzon D. (2010). Insight into invertebrate defensin mechanism of action: Oyster defensins inhibit peptidoglycan biosynthesis by binding to lipid II. J. Biol. Chem..

[114] Pushpanathan M., Gunasekaran P., Rajendhran J. (2013). Mechanisms of the antifungal action of marine metagenome-derived peptide, MMGP1, against *Candida albicans*. PLoS ONE.

[115] Cardoso M.H., de la Fuente-Nunez C., Santos N.C., Zasloff M.A., Franco O.L. (2024). Influence of antimicrobial peptides on the bacterial membrane curvature and vice versa. Trends Microbiol..

[116] Ramos-Martin F., D’Amelio N. (2022). Biomembrane lipids: When physics and chemistry join to shape biological activity. Biochimie.

[117] Arouri A., Dathe M., Blume A. (2013). The helical propensity of KLA amphipathic peptides enhances their binding to gel-state lipid membranes. Biophys. Chem..

[118] Marquette A., Bechinger B. (2018). Biophysical investigations elucidating the mechanisms of action of antimicrobial peptides and their synergism. Biomolecules.

[119] Lin B., Hung A., Singleton W., Darmawan K.K., Moses R., Yao B., Wu H., Barlow A., Marc-Antoine S., Sloan A.J. (2023). The effect of tailing lipidation on the bioactivity of antimicrobial peptides and their aggregation tendency. Aggregate.

[120] Porcelli F., Buck B., Lee D., Hallock K.J., Ramamoorthy A., Veglia G. (2004). Structure and orientation of pardaxin determined by NMR experiments in model membranes. J. Biol. Chem..

[121] Luo Y., Song Y. (2021). Mechanism of antimicrobial peptides: Antimicrobial, Anti-Inflammatory and antibiofilm activities. Int. J. Mol. Sci..

[122] Huan Y., Kong Q., Mou H., Yi H. (2020). Antimicrobial peptides: Classification, design, application and research progress in multiple fields. Front. Microbiol..

[123] Garcia-Fernandez R., Peigneur S., Pons T., Alvarez C., Gonzalez L., Chavez M.A., Tytgat J. (2016). The Kunitz-type protein ShPI-1 inhibits serine proteases and Voltage-Gated potassium channels. Toxins.

[124] Zhang K., Li X., Yu C., Wang Y. (2020). Promising therapeutic strategies against microbial biofilm challenges. Front. Cell. Infect. Microbiol..

[125] Flemming H., van Hullebusch E.D., Neu T.R., Nielsen P.H., Seviour T., Stoodley P., Wingender J., Wuertz S. (2023). The biofilm matrix: Multitasking in a shared space. Nat. Rev. Microbiol..

[126] Brancatisano F.L., Maisetta G., Di Luca M., Esin S., Bottai D., Bizzarri R., Campa M., Batoni G. (2014). Inhibitory effect of the human liver-derived antimicrobial peptide hepcidin 20 on biofilms of polysaccharide intercellular adhesin (PIA)-positive and PIA-negative strains of *Staphylococcus epidermidis*. Biofouling.

[127] Neshani A., Zare H., Akbari Eidgahi M.R., Khaledi A., Ghazvini K. (2019). Epinecidin-1, a highly potent marine antimicrobial peptide with anticancer and immunomodulatory activities. BMC Pharmacol. Toxicol..

[128] Chatterjee D., Sivashanmugam K. (2024). Immunomodulatory peptides: New therapeutic horizons for emerging and re-emerging infectious diseases. Front. Microbiol..

[129] Oh D., Strangman W.K., Kauffman C.A., Jensen P.R., Fenical W. (2007). Thalassospiramides a and b, immunosuppressive peptides from the marine bacterium *Thalassospira* sp.. Org. Lett..

[130] Wang Y., Sun L. (2022). A type ib crustin from deep-sea shrimp possesses antimicrobial and immunomodulatory activity. Int. J. Mol. Sci..

[131] Yao L., Yang P., Luo W., Li S., Wu Y., Cai N., Bi D., Li H., Han Q., Xu X. (2020). Macrophage-stimulating activity of European eel (*Anguilla anguilla*) peptides in RAW264.7 cells mediated via NF-kappaB and MAPK signaling pathways. Food Funct..

[132] Mallet J., Duarte J., Vinderola G., Anguenot R., Beaulieu M., Matar C. (2014). The immunopotentiating effects of shark-derived protein hydrolysate. Nutrition.

[133] Huang X., Mao W., Yi Y., Lu Y., Liu F., Deng L. (2024). The effects of four paralogous piscidin antimicrobial peptides on the chemotaxis, macrophage respiratory burst, phagocytosis and expression of immune-related genes in orange-spotted grouper (*Epinephelus coicodes*). Dev. Comp. Immunol..

[134] Gao X., Liu Y., Huang X., Yang Z., Sun M., Li F. (2025). A type Ia Crustin from the pacific white Shrimp *Litopenaeus vannamei* exhibits antimicrobial and chemotactic activities. Biomolecules.

[135] Zhang W., An Z., Bai Y., Zhou Y., Chen F., Wang K. (2023). A novel antimicrobial peptide Scyreptin(1–30) from *Scylla paramamosain* exhibiting potential therapy of *Pseudomonas aeruginosa* early infection in a mouse burn wound model. Biochem. Pharmacol..

[136] Garcia-Valtanen P., Martinez-Lopez A., Ortega-Villaizan M., Perez L., Coll J.M., Estepa A. (2014). In addition to its antiviral and immunomodulatory properties, the zebrafish beta-defensin 2 (zfBD2) is a potent viral DNA vaccine molecular adjuvant. Antiviral. Res..

[137] Amiss A.S., von Pein J.B., Webb J.R., Condon N.D., Harvey P.J., Phan M., Schembri M.A., Currie B.J., Sweet M.J., Craik D.J. (2021). Modified horseshoe crab peptides target and kill bacteria inside host cells. Cell. Mol. Life Sci..

[138] Pundir P., Catalli A., Leggiadro C., Douglas S.E., Kulka M. (2014). Pleurocidin, a novel antimicrobial peptide, induces human mast cell activation through the FPRL1 receptor. Mucosal Immunol..

[139] Li H., Niu J., Wang X., Niu M., Liao C. (2023). The contribution of antimicrobial peptides to immune cell function: A review of recent advances. Pharmaceutics.

[140] Diamond G., Beckloff N., Weinberg A., Kisich K.O. (2009). The roles of antimicrobial peptides in innate host defense. Curr. Pharm. Des..

[141] Bepler T., Barrera M.D., Rooney M.T., Xiong Y., Kuang H., Goodell E., Goodwin M.J., Harbron E., Fu R., Mihailescu M. (2024). Antiviral activity of the host defense peptide piscidin 1: Investigating a membrane-mediated mode of action. Front. Chem..

[142] Morimoto M., Mori H., Otake T., Ueba N., Kunita N., Niwa M., Murakami T., Iwanaga S. (1991). Inhibitory effect of tachyplesin I on the proliferation of human immunodeficiency virus in vitro. Chemotherapy.

[143] Narula P., Kiruthika S., Chowdhari S., Vivekanandan P., Chugh A. (2023). Inhibition of hepatitis b virus (HBV) by tachyplesin, a marine antimicrobial cell-penetrating peptide. Pharmaceutics.

[144] Xie H., Wei J., Qin Q. (2016). Antiviral function of tachyplesin I against iridovirus and nodavirus. Fish Shellfish Immunol..

[145] Plaza A., Gustchina E., Baker H.L., Kelly M., Bewley C.A. (2007). Mirabamides A-D, depsipeptides from the sponge *Siliquariaspongia mirabilis* that inhibit HIV-1 fusion. J. Nat. Prod..

[146] Martinez-Culebras P.V., Gandia M., Garrigues S., Marcos J.F., Manzanares P. (2021). Antifungal peptides and proteins to control toxigenic fungi and mycotoxin biosynthesis. Int. J. Mol. Sci..

[147] Pettit R.K., Pettit G.R., Hazen K.C. (1998). Specific activities of dolastatin 10 and peptide derivatives against *Cryptococcus neoformans*. Antimicrob. Agents. Chemother..

[148] Boyd M.R., Gustafson K.R., Mcmahon J.B., Shoemaker R.H., O’Keefe B.R., Mori T., Gulakowski R.J., Wu L., Rivera M.I., Laurencot C.M. (1997). Discovery of cyanovirin-N, a novel human immunodeficiency virus-inactivating protein that binds viral surface envelope glycoprotein gp120: Potential applications to microbicide development. Antimicrob. Agents. Chemother..

[149] Chiu F., Kuo H., Yu C., Selvam P., Su I., Tseng C., Yuan C., Wen Z. (2024). Marine-derived antimicrobial peptide piscidin-1 triggers extrinsic and intrinsic apoptosis in oral squamous cell carcinoma through reactive oxygen species production and inhibits angiogenesis. Free. Radic. Biol. Med..

[150] Zheng L., Li Y., Wang J., Pan Y., Chen J., Zheng W., Lin L. (2020). Antibacterial and antiparasitic activities analysis of a hepcidin—Like antimicrobial peptide from *Larimichthys crocea*. Acta Oceanol. Sin..

[151] Khalili S., Ebrahimzade E., Mohebali M., Shayan P., Mohammadi-Yeganeh S., Moghaddam M.M., Elikaee S., Akhoundi B., Sharifi-Yazdi M.K. (2019). Investigation of the antimicrobial activity of a short cationic peptide against promastigote and amastigote forms of *Leishmania* major (MHRO/IR/75/ER): An in vitro study. Exp. Parasitol..

[152] Mizuno Y., Makioka A., Kawazu S., Kano S., Kawai S., Akaki M., Aikawa M., Ohtomo H. (2002). Effect of jasplakinolide on the growth, invasion, and actin cytoskeleton of *Plasmodium falciparum*. Parasitol. Res..

[153] Makioka A., Kumagai M., Ohtomo H., Kobayashi S., Takeuchi T. (2000). Growth inhibition and actin aggregate formation of *Entamoeba histolytica* by jasplakinolide. Arch. Med. Res..

[154] Cruz G.S., Santos A.T.D., de Brito E.H.S., Radis-Baptista G. (2022). Cell-penetrating antimicrobial peptides with anti-infective activity against intracellular pathogens. Antibiotics.

[155] Zhang X., Cao D., Sun X., Sun S., Xu N. (2019). Preparation and identification of antioxidant peptides from protein hydrolysate of marine alga *Gracilariopsis lemaneiformis*. J. Appl. Phycol..

[156] Marinou D., Jacobsen C., Odelli D., Sarigiannidou K., Sorensen A.M. (2025). Production of protein hydrolysates from cod backbone using selected enzymes: Evaluation of antioxidative and antimicrobial activities of hydrolysates. Mar. Drugs.

[157] Liu Z., Li H., Li L., Ma Q., Fang Z., Wang H., Lee Y., Zhao J., Zhang H., Chen W. (2022). Gene-trait matching analysis reveals putative genes involved in *Bifidobacterium* spp. biofilm formation. Gene.

[158] Nowak A., Paliwoda A., Blasiak J. (2019). Anti-proliferative, pro-apoptotic and anti-oxidative activity of *Lactobacillus* and *Bifidobacterium* strains: A review of mechanisms and therapeutic perspectives. Crit. Rev. Food. Sci. Nutr..

[159] Ortizo R.G.G., Sharma V., Tsai M., Wang J., Sun P., Nargotra P., Kuo C., Chen C., Dong C. (2023). Extraction of novel bioactive peptides from fish protein hydrolysates by enzymatic reactions. Appl. Sci..

[160] Kim N.Y., Jung H.Y., Kim J.K. (2021). Identification and characterisation of a novel heptapeptide mackerel by-product hydrolysate, and its potential as a functional fertiliser component. J. Chromatogr. B.

[161] Han J., Huang Z., Tang S., Lu C., Wan H., Zhou J., Li Y., Ming T., Jim Wang Z., Su X. (2020). The novel peptides ICRD and LCGEC screened from tuna roe show antioxidative activity via Keap1/Nrf2-ARE pathway regulation and gut microbiota modulation. Food Chem..

[162] Chen J., Liang P., Xiao Z., Chen M., Gong F., Li C., Zhou C., Hong P., Jung W., Qian Z. (2019). Antiphotoaging effect of boiled abalone residual peptide ATPGDEG on UVB-induced keratinocyte HaCaT cells. Food Nutr. Res..

[163] Elsheikh M.A., Gaafar P.M.E., Khattab M.A., A Helwah M.K., Noureldin M.H., Abbas H. (2023). Dual-effects of caffeinated hyalurosomes as a nano-cosmeceutical gel counteracting UV-induced skin ageing. Int. J. Pharm. X.

[164] Chen T., Hou H. (2016). Protective effect of gelatin polypeptides from pacific cod (*Gadus macrocephalus*) against UV irradiation-induced damages by inhibiting inflammation and improving transforming growth factor-beta/smad signaling pathway. J. Photochem. Photobiol. B Biol..

[165] Sun L., Zhang Y., Zhuang Y. (2013). Antiphotoaging effect and purification of an antioxidant peptide from tilapia (*Oreochromis niloticus*) gelatin peptides. J. Funct. Foods.

[166] Robinson D.M., Vega J., Palm M.D., Bell M., Widgerow A.D., Giannini A. (2022). Multicenter evaluation of a topical hyaluronic acid serum. J. Cosmet. Dermatol..

[167] Juncan A.M., Moisa D.G., Santini A., Morgovan C., Rus L., Vonica-Tincu A.L., Loghin F. (2021). Advantages of hyaluronic acid and its combination with other bioactive ingredients in cosmeceuticals. Molecules.

[168] Kim H., Jeon B., Lee H., Chung D. (2020). Evaluation of the skin moisturizing efficacy of a collagen peptide isolated from fish scales, using HaCaT keratinocytes. J. Korean Soc. Food Sci. Nutr..

[169] Jo D., Khan F., Park S., Ko S., Kim K.W., Yang D., Kim J., Oh G., Choi G., Lee D. (2024). From sea to lab: Angiotensin I-converting enzyme inhibition by marine peptides—Mechanisms and applications. Mar. Drugs.

[170] Purcell D., Packer M.A., Hayes M. (2022). Angiotensin-I-converting enzyme inhibitory activity of protein hydrolysates generated from the macroalga *Laminaria digitata* (Hudson) JV Lamouroux 1813. Foods.

[171] Cao J., Xiang B., Dou B., Hu J., Zhang L., Kang X., Lyu M., Wang S. (2024). Novel angiotensin-converting enzyme-inhibitory peptides obtained from *Trichiurus lepturus*: Preparation, identification and potential antihypertensive mechanism. Biomolecules.

[172] Nasab S.B., Homaei A., Pletschke B., Salinas-Salazar C., Castillo-Zacarias C., Parra-Saldivar R. (2020). Marine resources effective in controlling and treating diabetes and its associated complications. Process Biochem..

[173] Harnedy P.A., Parthsarathy V., Mclaughlin C.M., O’Keeffe M.B., Allsopp P.J., Mcsorley E.M., O’Harte F.P.M., Fitzgerald R.J. (2018). Atlantic salmon (salmo salar) co-product-derived protein hydrolysates: A source of antidiabetic peptides. Food Res. Int..

[174] Wright J.R.J. (2021). Frederick Banting’s actual great idea: The role of fetal bovine islets in the discovery of insulin. Islets.

[175] Mitta G., Hubert F., Noel T., Roch P. (1999). Myticin, a novel cysteine-rich antimicrobial peptide isolated from haemocytes and plasma of the mussel *Mytilus galloprovincialis*. Eur. J. Biochem..

[176] Ojeda P.G., Cardoso M.H., Franco O.L. (2019). Pharmaceutical applications of cyclotides. Drug Discov. Today.

[177] Zhang H., Chen S. (2022). Cyclic peptide drugs approved in the last two decades (2001–2021). RSC Chem. Biol..

[178] Dong S., Song H., Zhao Y., Liu Z., Wei B., Zeng M. (2012). The preparation and antimicrobial activity of peptide fractions from blue mussel (*Mytilus edulis*) protein hydrolysate. Adv. Mater. Res..

[179] Abuine R., Rathnayake A.U., Byun H. (2019). Biological activity of peptides purified from fish skin hydrolysates. Fish. Aquat. Sci..

[180] Yusof F., Chowdhury S., Faruck M.O., Sulaiman N. (2017). Anticancer peptides derived from supermeal worm (*Zophobas morio*) larvae. Int. Food Res. J..

[181] Seyedjavadi S.S., Razzaghi-Abyaneh M., Nasiri M.J., Hashemi A., Goudarzi H., Haghighi M., Dadashi M., Goudarzi M., Zare-Zardini H., Pourhossein B. (2022). Isolation and chemical characterization of an alpha-helical peptide, Dendrocin-ZM1, derived from *Zataria multiflora* boiss with potent antibacterial activity. Probiotics Antimicrob. Proteins.

[182] Tang K.H.D., Lock S.S.M., Yap P., Cheah K.W., Chan Y.H., Yiin C.L., Ku A.Z.E., Loy A.C.M., Chin B.L.F., Chai Y.H. (2022). Immobilized enzyme/microorganism complexes for degradation of microplastics: A review of recent advances, feasibility and future prospects. Sci. Total Environ..

[183] Dawson P.L., Harmon L., Sotthibandhu A., Han I.Y. (2005). Antimicrobial activity of nisin-adsorbed silica and corn starch powders. Food Microbiol..

[184] Patrulea V., Borchard G., Jordan O. (2020). An update on antimicrobial peptides (AMPs) and their delivery strategies for wound infections. Pharmaceutics.

[185] Lee H., Lim S.I., Shin S., Lim Y., Koh J.W., Yang S. (2019). Conjugation of cell-penetrating peptides to antimicrobial peptides enhances antibacterial activity. ACS Omega.

[186] Armstrong M.J., Gaunt P., Aithal G.P., Barton D., Hull D., Parker R., Hazlehurst J.M., Guo K., Abouda G., Aldersley M.A. (2016). Liraglutide safety and efficacy in patients with non-alcoholic steatohepatitis (LEAN): A multicentre, double-blind, randomised, placebo-controlled phase 2 study. Lancet.

[187] Ferrazzano L., Catani M., Cavazzini A., Martelli G., Corbisiero D., Cantelmi P., Fantoni T., Mattellone A., De Luca C., Felletti S. (2022). Sustainability in peptide chemistry: Current synthesis and purification technologies and future challenges. Green Chem..

[188] Berillo D., Al-Jwaid A., Caplin J. (2021). Polymeric materials used for immobilisation of bacteria for the bioremediation of contaminants in water. Polymers.

[189] Kanaujia K.A., Wagh S., Pandey G., Phatale V., Khairnar P., Kolipaka T., Rajinikanth P.S., Saraf S.A., Srivastava S., Kumar S. (2025). Harnessing marine antimicrobial peptides for novel therapeutics: A deep dive into ocean-derived bioactives. Int. J. Biol. Macromol..

[190] Varnava K.G., Sarojini V. (2019). Making Solid-phase peptide synthesis greener: A review of the literature. Chem.-Asian J..

[191] Hansen A.M., Bonke G., Hogendorf W.F.J., Bjorkling F., Nielsen J., Kongstad K.T., Zabicka D., Tomczak M., Urbas M., Nielsen P.E. (2019). Microwave-assisted solid-phase synthesis of antisense acpP peptide nucleic acid-peptide conjugates active against colistin-and tigecycline-resistant *E. coli* and *K. pneumoniae*. Eur. J. Med. Chem..

[192] Chen J., Sun S., Zhao R., Xi C., Qiu W., Wang N., Wang Y., Bierer D., Shi J., Li Y. (2020). Chemical synthesis of six-atom thioether bridged diaminodiacid for solid-phase synthesis of peptide disulfide bond mimics. ChemistrySelect.

[193] Ozawa A., Cai Y., Lindberg I. (2007). Production of bioactive peptides in an in vitro system. Anal. Biochem..

[194] Brookwell A., Oza J.P., Caschera F. (2021). Biotechnology applications of cell-free expression systems. Life.

[195] Pardee K., Slomovic S., Nguyen P.Q., Lee J.W., Donghia N., Burrill D., Ferrante T., Mcsorley F.R., Furuta Y., Vernet A. (2016). Portable, on-demand biomolecular manufacturing. Cell.

[196] Jin X., Kightlinger W., Kwon Y., Hong S.H. (2018). Rapid production and characterization of antimicrobial colicins using *Escherichia coli*-based cell-free protein synthesis. Synth. Biol..

[197] Yang N., Wang X., Teng D., Mao R., Hao Y., Zong L., Feng X., Wang J. (2016). Modification and characterization of a new recombinant marine antimicrobial peptide N2. Process Biochem..

[198] Li X., Jiang Y., Lin Y. (2022). Production of antimicrobial peptide arasin-like Sp in *Escherichia coli* via an ELP-intein self-cleavage system. J. Biotechnol..

[199] Neshani A., Eidgahi M.R.A., Zare H., Ghazvini K. (2018). Extended-spectrum antimicrobial activity of the low cost produced tilapia piscidin 4 (TP4) marine antimicrobial peptide. J. Res. Med. Dent. Sci..

[200] Zhao Q., Yang N., Gu X., Li Y., Teng D., Hao Y., Lu H., Mao R., Wang J. (2024). High-Yield preparation of american oyster defensin (AOD) via a small and acidic fusion tag and its functional characterization. Mar. Drugs.

[201] Bryksa B.C., Macdonald L.D., Patrzykat A., Douglas S.E., Mattatall N.R. (2006). A C-terminal glycine suppresses production of pleurocidin as a fusion peptide in *Escherichia coli*. Protein. Expr. Purif..

[202] Gaglione R., Pane K., Dell’Olmo E., Cafaro V., Pizzo E., Olivieri G., Notomista E., Arciello A. (2019). Cost-effective production of recombinant peptides in *Escherichia coli*. New Biotech..

[203] Wei Q.D., Kim Y.S., Seo J.H., Cha H.J. (2005). Facilitation of expression and purification of an antimicrobial peptide by fusion with baculoviral polyhedrin in *Escherichia coli*. Appl. Environ. Microbiol..

[204] Zhu S., Weber D.K., Separovic F., Sani M. (2021). Expression and purification of the native c-amidated antimicrobial peptide Maculatin 1.1. J. Pept. Sci..

[205] Lee B., Tsai J., Lin C., Hung C., Sheu J., Tsai H. (2021). Using *Bacillus subtilis* as a host cell to express an antimicrobial peptide from the marine chordate *Ciona intestinalis*. Mar. Drugs.

[206] Meng D., Zhao J., Ling X., Dai H., Guo Y., Gao X., Dong B., Zhang Z., Meng X., Fan Z. (2017). Recombinant expression, purification and antimicrobial activity of a novel antimicrobial peptide PaDef in *Pichia pastoris*. Protein. Expr. Purif..

[207] Garvey M. (2022). Non-Mammalian eukaryotic expression systems yeast and fungi in the production of biologics. J. Fungi.

[208] Dong C., Li M., Zhang R., Lu W., Xu L., Liu J., Chu X. (2023). The expression of antibacterial peptide turgencin a in *Pichia pastoris* and an analysis of its antibacterial activity. Molecules.

[209] Chiou M., Chen L., Peng K., Pan C., Lin T., Chen J. (2009). Stable expression in a Chinese hamster ovary (CHO) cell line of bioactive recombinant chelonianin, which plays an important role in protecting fish against pathogenic infection. Dev. Comp. Immunol..

[210] Felberbaum R.S. (2015). The baculovirus expression vector system: A commercial manufacturing platform for viral vaccines and gene therapy vectors. Biotechnol. J..

[211] He Y., Peng H., Liu J., Chen F., Zhou Y., Ma X., Chen H., Wang K. (2018). Transgenic with Scy-hepc enhancing the survival of *Sparus macrocephalus* and hybrid grouper challenged with *Aeromonas hydrophila*. Fish Shellfish Immunol..

[212] Shanmugaraj B., Bulaon C.J.I., Phoolcharoen W. (2020). Plant molecular farming: A viable platform for recombinant biopharmaceutical production. Plants.

[213] Bock R. (2015). Engineering plastid genomes: Methods, tools, and applications in basic research and biotechnology. Annu. Rev. Plant Biol..

[214] Chaudhary S., Ali Z., Mahfouz M. (2024). Molecular farming for sustainable production of clinical-grade antimicrobial peptides. Plant Biotechnol. J..

[215] Chaudhary S., Mahfouz M.M. (2024). Molecular farming of antimicrobial peptides. Nat. Rev. Bioeng..

[216] Jaiswal M., Singh A., Kumar S. (2023). PTPAMP: Prediction tool for plant-derived antimicrobial peptides. Amino Acids.

[217] Wang K., Gao Z., Wang Y., Meng C., Li J., Qin S., Cui Y. (2021). The chloroplast genetic engineering of a unicellular green alga chlorella vulgaris with two foreign peptides Co-Expression. Algal Res..

[218] Chai T., Tan Y., Ee K., Xiao J., Wong F. (2019). Seeds, fermented foods, and agricultural by-products as sources of plant-derived antibacterial peptides. Crit. Rev. Food. Sci. Nutr..

[219] Lee I.H., Zhao C., Cho Y., Harwig S.S., Cooper E.L., Lehrer R.I. (1997). Clavanins, alpha-helical antimicrobial peptides from tunicate hemocytes. FEBS Lett..

[220] Fan L., Wei Y., Chen Y., Jiang S., Xu F., Zhang C., Wang H., Shao X. (2023). Epinecidin-1, a marine antifungal peptide, inhibits *Botrytis cinerea* and delays gray mold in postharvest peaches. Food Chem..

[221] Bi J., Tian C., Jiang J., Zhang G., Hao H., Hou H. (2020). Antibacterial activity and potential application in food packaging of peptides derived from turbot viscera hydrolysate. J. Agric. Food. Chem..

[222] Liu Z., Zeng M., Dong S., Xu J., Song H., Zhao Y. (2007). Effect of an antifungal peptide from oyster enzymatic hydrolysates for control of gray mold (*Botrytis cinerea*) on Harvested Strawberries. Postharvest Biol. Technol..

[223] Tassanakajon A., Amparyup P., Somboonwiwat K., Supungul P. (2010). Cationic antimicrobial peptides in penaeid shrimp. Mar. Biotechnol..

[224] Lin Y., Yang S., Wang X., Xie R., Cheng J., He T., Chen X., Zhang X. (2022). A synthetic peptide based on large yellow croaker (*Larimichthys crocea*) IFNG1R protein sequence has potential antimicrobial activity against *Pseudomonas plecoglossicida*. Front. Mar. Sci..

[225] Phuket T.R.N., Charoensapsri W., Amparyup P., Imjongjirak C. (2023). Antibacterial activity and immunomodulatory role of a proline-rich antimicrobial peptide SpPR-AMP1 against *Vibrio campbellii* infection in shrimp *Litopenaeus vannamei*. Fish Shellfish Immunol..

[226] Huang H., Su B., Tsai T., Rajanbabu V., Pan C., Chen J. (2020). Dietary supplementation of recombinant tilapia piscidin 4-expressing yeast enhances growth and immune response in Lates calcarifer. Aquacult. Rep..

[227] An Z., Chen F., Hao H., Xiong M., Peng H., Sun H., Wang K. (2023). Growth-promoting effect of antimicrobial peptide Scy-hepc on mariculture large yellow croaker *Larimichthys crocea* and the underlying mechanism. Fish Shellfish Immunol..

[228] Doiron K., Beaulieu L., St-Louis R., Lemarchand K. (2018). Reduction of bacterial biofilm formation using marine natural antimicrobial peptides. Colloid Surf. B-Biointerfaces.

[229] Han J., Jyoti M.A., Song H., Jang W.S. (2016). Antifungal activity and action mechanism of histatin 5-halocidin hybrid peptides against *Candida* ssp. PLoS ONE.

[230] Jang W.S., Kim H.K., Lee K.Y., Kim S.A., Han Y.S., Lee I.H. (2006). Antifungal activity of synthetic peptide derived from halocidin, antimicrobial peptide from the tunicate, *Halocynthia aurantium*. FEBS Lett..

[231] Nixon A.E., Sexton D.J., Ladner R.C. (2014). Drugs derived from phage display: From candidate identification to clinical practice. MAbs.

[232] Hong J., Hu J., Ke F. (2016). Experimental induction of bacterial resistance to the antimicrobial peptide tachyplesin I and investigation of the resistance mechanisms. Antimicrob. Agents. Chemother..

[233] Guo Z., Hou Y., Tian Y., Tian J., Hu J., Zhang Y. (2024). Antimicrobial peptide hydrogel with pH-responsive and controllable drug release properties for the efficient treatment of *helicobacter pylori* infection. ACS Appl. Mater. Interfaces.

[234] Starr C.G., He J., Wimley W.C. (2016). Host cell interactions are a significant barrier to the clinical utility of peptide antibiotics. ACS Chem. Biol..

[235] Gruden S., Ulrih N.P. (2021). Diverse mechanisms of antimicrobial activities of lactoferrins, lactoferricins, and other lactoferrin-derived peptides. Int. J. Mol. Sci..

[236] Alfei S., Schito A.M. (2020). Positively charged polymers as promising devices against multidrug resistant gram-negative bacteria: A review. Polymers.

[237] Dong L., Li Y., Zhang Y., Su S. (2025). Cationic antimicrobial peptide CC34 potential anticancer and apoptotic induction on cancer cells. Amino Acids.

[238] Ma S., Li S., Song L., Riaz A., Huang R., Zhou S., Qiu J., Chu Z., He J. (2025). Isolation and synthesis of a new cyclic tetrapeptide from marine-associated *Bacillus* sp. and its bacterial biofilm formation inhibitory activity. J. Antibiot..

[239] Yakir I., Cohen E., Schlesinger S., Hayouka Z. (2025). Random antimicrobial peptide mixtures as non-antibiotic antimicrobial agents for cultured meat industry. Food Chem. Mol. Sci..

[240] Tincu J.A., Menzel L.P., Azimov R., Sands J., Hong T., Waring A.J., Taylor S.W., Lehrer R.I. (2003). Plicatamide, an antimicrobial octapeptide from *Styela Plicata* Hemocytes. J. Biol. Chem..

[241] Panteleev P.V., Tsarev A.V., Bolosov I.A., Paramonov A.S., Marggraf M.B., Sychev S.V., Shenkarev Z.O., Ovchinnikova T.V. (2018). Novel antimicrobial peptides from the arctic polychaeta *Nicomache minor* provide new molecular insight into biological role of the BRICHOS Domain. Mar. Drugs.

[242] Marr A.K., Gooderham W.J., Hancock R.E. (2006). Antibacterial peptides for therapeutic use: Obstacles and realistic outlook. Curr. Opin. Pharmacol..

[243] Jin Q., Peng D., Zheng Z. (2022). Advances in extracting and understanding the bioactivities of marine organism peptides: A review. J. Food Process Preserv..

[244] Guyonnet D., Fremaux C., Cenatiempo Y., Berjeaud J.M. (2000). Method for rapid purification of class IIa bacteriocins and comparison of their activities. Appl. Environ. Microbiol..

[245] Chumchalova J., Stiles J., Josephsen J., Plockova M. (2004). Characterization and purification of acidocin CH5, a bacteriocin produced by *Lactobacillus acidophilus* CH5. J. Appl. Microbiol..

[246] Sarkar T., Chetia M., Chatterjee S. (2021). Antimicrobial peptides and proteins: From nature’s reservoir to the laboratory and beyond. Front. Chem..

[247] Deptula M., Wardowska A., Dzierzynska M., Rodziewicz-Motowidlo S., Pikula M. (2018). Antibacterial peptides in dermatology-strategies for evaluation of allergic potential. Molecules.

[248] Costa F., Teixeira C., Gomes P., Martins M.C.L. (2019). Clinical application of AMPs. Adv. Exp. Med. Biol..

[249] Chen C.H., Lu T.K. (2020). Development and challenges of antimicrobial peptides for therapeutic applications. Antibiotics.

[250] Ma X., Aminov R., Franco O.L., de la Fuente-Nunez C., Wang G., Wang J. (2024). Editorial: Antimicrobial peptides and their druggability, bio-safety, stability, and resistance. Front. Microbiol..

[251] Patra A., Das J., Agrawal N.R., Kushwaha G.S., Ghosh M., Son Y. (2022). Marine antimicrobial peptides-based strategies for tackling bacterial biofilm and biofouling challenges. Molecules.

[252] Przeslawski R., Foster S.D. (2020). Field Manuals for Marine Sampling in Australian Waters.

[253] Schroeder C.I., Lewis R.J. (2006). Ω-conotoxins GVIA, MVIIA and CVID: SAR and clinical potential. Mar. Drugs.

[254] Santhiravel S., Dave D., Shahidi F. (2025). Bioactives from marine resources as natural health products: A review. Pharmacol. Rev..

[255] Deng C., Wu J., Cheng R., Meng F., Klok H., Zhong Z. (2014). Functional polypeptide and hybrid materials: Precision synthesis via α-amino acid N-carboxyanhydride polymerization and emerging biomedical applications. Prog. Polym. Sci..

[256] Pan H., Ye R., Han S., Li A., Zhou Y., Li Y., Yang D., Lin J., Dai H., Dang X. (2025). Discovery of a novel sea snake antimicrobial peptide Hydrostatin-AMP3 with dual-mechanism against multidrug-resistant *Klebsiella pneumoniae*. Eur. J. Med. Chem..

[257] Chen S., Liu D., Wang L., Fan A., Wu M., Xu N., Zhu K., Lin W. (2025). Marine-derived new peptaibols with antibacterial activities by targeting bacterial membrane phospholipids. Acta Pharm. Sin. B.

[258] Hsu H., Chen M., Yeh M., Chen W. (2022). Antibacterial and anticancer activities of pleurocidin-amide, a potent marine antimicrobial peptide derived from winter flounder, *Pleuronectes americanus*. Mar. Drugs.

[259] Ramezanzade L., Hosseini S.F., Nikkhah M. (2017). Biopolymer-coated nanoliposomes as carriers of rainbow trout skin-derived antioxidant peptides. Food Chem..

[260] Sun H., Hong Y., Xi Y., Zou Y., Gao J., Du J. (2018). Synthesis, Self-Assembly, and biomedical applications of antimicrobial peptide-polymer conjugates. Biomacromolecules.

[261] Lepeltier E., Rijo P., Rizzolio F., Popovtzer R., Petrikaite V., Assaraf Y.G., Passirani C. (2020). Nanomedicine to target multidrug resistant tumors. Drug Resist. Update.

[262] Zhang C., Chen F., Bai Y., Dong X., Meng X., Wang K. (2024). A novel antimicrobial peptide Spasin141-165 identified from *Scylla paramamosain* exhibiting protection against *Aeromonas hydrophila* infection. Aquaculture.

[263] Kesavan D., Philip R. (2025). Unveiling the modes of action of a recombinant antimicrobial peptide, hepcidin (rGf-Hep), from *Gerres filamentosus* against pathogenic vibrios: Membrane disintegration and reactive oxygen species generation leading to cell death. Probiotics Antimicrob. Proteins.

[264] Hosseini S.F., Ramezanzade L., Mcclements D.J. (2021). Recent advances in nanoencapsulation of hydrophobic marine bioactives: Bioavailability, safety, and sensory attributes of nano-fortified functional foods. Trends Food Sci. Technol..

